# Supplementary material for: Elucidating trends and underlying drivers of neonatal mortality stagnation in Nepal: An analytical perspective on the 2016 and 2022 Demographic and Health Surveys
Source: PLoS One. 2025 Aug 22;20(8):e0330734. doi: 10.1371/journal.pone.0330734 (PMC12373174; doi:10.1371/journal.pone.0330734)
Supplement: S1 Table — (DOCX) [file pone.0330734.s001.docx]

S1 Table: The Numbers of Births; the Numbers of Early Neonatal, Late Neonatal, and Neonatal Deaths; and the Early Neonatal, Late Neonatal, and NMRs per 1,000 Births, for all Categories of the Covariates, for the Five Years Before the 2016 NDHS.

| **Characteristics** | **Categories** | **Births** | **Early ND** | **Late ND** | **ND** | **Early NMR** | **Late NMR** | **NMR** |
| --- | --- | --- | --- | --- | --- | --- | --- | --- |
| National | National | 5,087 | 84 | 22 | 106 | 16.6 | 4.3 | 20.9 |
| **Household characteristics** | | | | | | | | |
| Respondent’s language | Bhojpuri | 707 | 12 | 6 | 18 | 17.6 | 8.2 | 25.8 |
|  | Maithili | 920 | 16 | 6 | 22 | 16.9 | 6.5 | 23.4 |
|  | Nepali | 2,128 | 31 | 6 | 37 | 14.7 | 2.6 | 17.4 |
|  | Other | 1,332 | 25 | 5 | 30 | 18.8 | 3.5 | 22.3 |
| Ethnicity (three categories) | Advantaged | 2,615 | 38 | 11 | 48 | 14.4 | 4 | 18.4 |
|  | Disadvantaged Dalit | 1,065 | 22 | 6 | 29 | 21.1 | 5.9 | 27 |
|  | Disadvantaged Janajati | 1,407 | 24 | 5 | 29 | 17.3 | 3.6 | 20.9 |
| Ethnicity (two categories) | Advantaged | 1,600 | 24 | 5 | 29 | 15 | 3.3 | 18.3 |
|  | Disadvantaged | 3,487 | 60 | 17 | 77 | 17.3 | 4.8 | 22.1 |
| Wealth index in terciles | Poorer | 1,412 | 27 | 7 | 34 | 19.3 | 4.8 | 24.1 |
|  | Middle | 1,806 | 33 | 6 | 39 | 18.2 | 3.2 | 21.4 |
|  | Higher | 1,869 | 24 | 9 | 34 | 13 | 5 | 18 |
| Wealth index | Middle | 1,124 | 15 | 3 | 18 | 13.4 | 2.4 | 15.8 |
|  | Poorer and poorest | 2,163 | 48 | 11 | 59 | 22.1 | 5.2 | 27.3 |
|  | Richer and richest | 1,799 | 21 | 8 | 29 | 11.9 | 4.5 | 16.4 |
| Province | Koshi | 816 | 14 | 5 | 19 | 17.1 | 5.8 | 22.8 |
|  | Madhesh | 1,376 | 24 | 10 | 33 | 17.3 | 7 | 24.3 |
|  | Bagmati | 825 | 9 | 0 | 9 | 10.9 |  | 10.9 |
|  | Gandaki | 390 | 4 | 1 | 5 |  |  |  |
|  | Lumbini | 897 | 13 | 4 | 17 | 14.1 | 4.3 | 18.4 |
|  | Karnali | 341 | 9 | 2 | 11 | 26.4 | 4.9 | 31.3 |
|  | Sudurpaschim | 441 | 12 | 1 | 13 | 26.7 | 3.2 | 29.9 |
| Ecological region | Hill | 1,918 | 28 | 4 | 31 | 14.5 | 1.8 | 16.4 |
|  | Mountain | 360 | 10 | 3 | 13 | 28.5 | 7.9 | 36.5 |
|  | Terai | 2,809 | 46 | 16 | 62 | 16.4 | 5.6 | 22 |
| Religion | Buddhist | 217 | 2 | 0 | 2 | 7.3 |  | 7.3 |
|  | Hindu | 4,337 | 77 | 20 | 98 | 17.8 | 4.7 | 22.5 |
|  | Muslim | 362 | 5 | 2 | 7 | 15.2 | 4.3 | 19.4 |
|  | Other | 170 | 0 | 0 | 0 |  |  |  |
| Type of place | Rural | 2,335 | 50 | 11 | 61 | 21.5 | 4.8 | 26.3 |
|  | Urban | 2,752 | 34 | 11 | 45 | 12.4 | 4 | 16.3 |
| Size of household | <Six members | 2,399 | 43 | 8 | 51 | 18 | 3.3 | 21.2 |
|  | ≥Six members | 2,688 | 41 | 14 | 55 | 15.3 | 5.3 | 20.6 |
| Sex of household head | Female | 1,470 | 23 | 2 | 25 | 15.7 | 1.6 | 17.3 |
|  | Male | 3,617 | 61 | 20 | 81 | 16.9 | 5.4 | 22.4 |
| Indoor air pollution | No | 1,571 | 19 | 9 | 27 | 11.8 | 5.6 | 17.4 |
|  | Yes | 3,516 | 66 | 13 | 79 | 18.7 | 3.8 | 22.5 |
| Improved water and sanitation | Improved | 3,334 | 55 | 11 | 65 | 16.4 | 3.2 | 19.6 |
|  | Not a de jure resident | 386 | 10 | 4 | 15 | 26.4 | 11.6 | 38 |
|  | Unimproved | 1,367 | 19 | 7 | 26 | 14.3 | 5 | 19.3 |
| **Maternal-related characteristics** | | | | | | | | |
| Maternal education | Basic (grades 1–8) | 1,025 | 23 | 1 | 24 | 22.1 | 1.3 | 23.3 |
|  | No education | 1,761 | 35 | 11 | 45 | 19.6 | 6.1 | 25.7 |
|  | Secondary and above (≥grade nine) | 2,301 | 27 | 10 | 37 | 11.8 | 4.3 | 16.1 |
| Maternal age (five categories) | 15–19 years | 369 | 11 | 2 | 12 | 29 | 4.2 | 33.2 |
|  | 20–24 years | 1,662 | 33 | 13 | 46 | 19.7 | 8 | 27.7 |
|  | 25–29 years | 1,805 | 24 | 5 | 29 | 13.3 | 2.7 | 16 |
|  | 30–34 years | 824 | 11 | 1 | 12 | 13.4 | 1.6 | 15 |
|  | 35 and above | 426 | 6 | 1 | 7 | 13.4 | 2.7 | 16.1 |
| Maternal age (three categories) | 15–19 years | 369 | 11 | 2 | 12 | 29 | 4.2 | 33.2 |
|  | 20–34 years | 4,292 | 68 | 19 | 87 | 15.8 | 4.5 | 20.3 |
|  | ≥35 years | 426 | 6 | 1 | 7 | 13.4 | 2.7 | 16.1 |
| Maternal use of tobacco | No | 4,796 | 81 | 21 | 102 | 16.8 | 4.5 | 21.3 |
|  | Yes | 291 | 4 | 1 | 4 | 12.4 | 2.1 | 14.5 |
| Maternal stature | <145 cm | 279 | 4 | 4 | 8 | 14.4 | 14.7 | 29.2 |
|  | ≥145 cm | 2,271 | 44 | 7 | 51 | 19.4 | 2.9 | 22.3 |
| Maternal anemia | Anemic | 1,153 | 28 | 5 | 33 | 24.4 | 4.6 | 29 |
|  | Not anemic | 1,387 | 19 | 5 | 25 | 14 | 3.9 | 17.8 |
| **Empowerment-related characteristics** | | | | | | | | |
| Owns a mobile phone | No | 1,172 | 23 | 11 | 34 | 19.6 | 9.2 | 28.8 |
|  | Yes | 3,915 | 61 | 11 | 73 | 15.7 | 2.9 | 18.5 |
| Possess a bank account | No | 3,284 | 57 | 19 | 75 | 17.2 | 5.7 | 22.9 |
|  | Yes | 1,803 | 28 | 3 | 31 | 15.3 | 1.9 | 17.2 |
| Internet use | Never used Internet | 4,149 | 74 | 20 | 94 | 17.8 | 4.8 | 22.5 |
|  | Used at some time | 938 | 11 | 2 | 13 | 11.2 | 2.5 | 13.7 |
| Empowerment: household decisions | No | 3,131 | 57 | 14 | 71 | 18.1 | 4.6 | 22.7 |
|  | Yes, can make decisions | 1,956 | 28 | 8 | 35 | 14.2 | 3.9 | 18 |
| Violence justified | Violence is not justified | 3,621 | 58 | 14 | 72 | 16.1 | 3.8 | 19.9 |
|  | Violence is justified | 1,466 | 26 | 8 | 34 | 17.6 | 5.6 | 23.3 |
| Empowerment: health care/family planning decisions | No | 4,116 | 79 | 17 | 96 | 19.3 | 4.1 | 23.4 |
|  | Yes | 971 | 5 | 5 | 10 | 5.1 | 5.1 | 10.2 |
| Newspaper/Magazine | At least once a week | 2,713 | 41 | 12 | 53 | 15 | 4.4 | 19.4 |
|  | Less than once a week | 2,374 | 43 | 10 | 54 | 18.3 | 4.3 | 22.6 |
| Radio/TV | Less than once a week | 2,385 | 43 | 10 | 54 | 18.2 | 4.2 | 22.5 |
|  | At least once a week | 2,702 | 41 | 12 | 53 | 15.1 | 4.4 | 19.5 |
| Knows about HMG | No | 3,405 | 58 | 16 | 74 | 17 | 4.6 | 21.7 |
|  | Yes | 1,682 | 26 | 6 | 33 | 15.6 | 3.8 | 19.4 |
| **Husband’s characteristics** | | | | | | | | |
| Husband’s education | Basic (grades 1–8) | 1,155 | 32 | 3 | 35 | 27.3 | 2.6 | 29.9 |
|  | No education/Do not know | 772 | 11 | 4 | 15 | 14.6 | 4.8 | 19.5 |
|  | Secondary and above (≥grade nine) | 3,122 | 41 | 15 | 57 | 13.3 | 4.9 | 18.2 |
| Husband’s occupation (four categories) | Agriculture | 954 | 23 | 6 | 29 | 23.9 | 6.7 | 30.6 |
|  | Manual (skilled/unskilled) | 2,019 | 36 | 6 | 42 | 17.6 | 3 | 20.7 |
|  | Not working | 161 | 0 | 0 | 0 |  |  |  |
|  | Sales, clerical, other | 1,915 | 26 | 10 | 35 | 13.5 | 5 | 18.5 |
| **Birth characteristics** | | | | | | | | |
| Birthweight taken | Not taken | 1,941 | 58 | 6 | 64 | 30 | 3.2 | 33.2 |
|  | Yes, taken | 3,048 | 24 | 15 | 38 | 7.8 | 4.8 | 12.5 |
| Sex of child | Female | 2,426 | 35 | 7 | 42 | 14.3 | 3 | 17.3 |
|  | Male | 2,661 | 50 | 15 | 64 | 18.6 | 5.5 | 24.2 |
| Birthweight | Large (≥3,500 g) | 901 | 7 | 3 | 10 | 7.9 | 3.1 | 11 |
|  | Normal (2,500 g–3,500 g) | 1,768 | 7 | 10 | 17 | 4.2 | 5.6 | 9.8 |
|  | Not weighed or do not know | 1,941 | 58 | 6 | 64 | 30 | 3.2 | 33.2 |
|  | Small (<2,500 g) | 376 | 8 | 2 | 10 | 22.2 | 5 | 27.2 |
| Perceived birthweight | Very large | 175 | 5 | 0 | 5 | 26.5 |  | 26.5 |
|  | Larger than average | 646 | 15 | 5 | 21 | 23.7 | 8.5 | 32.2 |
|  | Average | 3,306 | 36 | 8 | 43 | 10.8 | 2.3 | 13 |
|  | Smaller than average | 622 | 15 | 7 | 22 | 23.5 | 11.5 | 34.9 |
|  | Very small | 230 | 8 | 1 | 8 | 32.8 | 2.5 | 35.2 |
|  | ’Do not know | 10 | 4 | 0 | 4 | 411.1 |  | 411.1 |
| **Pregnancy-related characteristics** | | | | | | | | |
| Birth order | First born | 2,000 | 40 | 9 | 49 | 19.9 | 4.4 | 24.3 |
|  | 2–4 | 2,653 | 35 | 11 | 47 | 13.2 | 4.3 | 17.6 |
|  | Five or more | 434 | 9 | 2 | 11 | 21.4 | 4 | 25.5 |
| Mother’s parity | Primigravida | 2,688 | 53 | 16 | 69 | 19.6 | 6 | 25.6 |
|  | Multigravida | 2,399 | 32 | 6 | 38 | 13.2 | 2.5 | 15.7 |
| Preceding birth interval | >Two years | 2,324 | 25 | 9 | 34 | 10.8 | 3.8 | 14.6 |
|  | First birth | 2,000 | 40 | 9 | 49 | 19.9 | 4.4 | 24.3 |
|  | ≤Two years | 754 | 18 | 4 | 22 | 23.7 | 5.8 | 29.4 |
| Twin birth | No | 5,023 | 79 | 22 | 101 | 15.7 | 4.4 | 20.1 |
|  | Yes | 30 | 2 | 0 | 2 | 74.3 |  | 74.3 |
| Wanted last birth | Wanted then | 3,920 | 66 | 17 | 83 | 17 | 4.3 | 21.2 |
|  | Wanted later | 655 | 10 | 1 | 12 | 15.6 | 2.1 | 17.7 |
|  | Wanted no more | 463 | 8 | 4 | 12 | 16.4 | 8.5 | 24.9 |
| **Health system factors** | | | | | | | | |
| Time to health facility | <=30 minutes | 1,156 | 2 | 3 | 5 | 1.8 | 2.4 | 4.2 |
|  | >30 minutes | 1,096 | 10 | 1 | 11 | 9.2 | 0.6 | 9.8 |
| Birth attendants | Delivery without SBA | 2,102 | 47 | 7 | 54 | 22.6 | 3.1 | 25.7 |
|  | Delivery with SBA | 2,887 | 34 | 14 | 49 | 11.9 | 4.9 | 16.9 |
| Place of delivery | Home delivery | 2,074 | 46 | 8 | 54 | 22.3 | 3.6 | 25.9 |
|  | Public health facility | 2,147 | 24 | 8 | 32 | 11.1 | 3.9 | 14.9 |
|  | Private health facility | 768 | 12 | 5 | 17 | 15.5 | 6.4 | 21.9 |
| C-section past delivery | Caesarean | 450 | 2 | 2 | 4 | 4.6 | 3.7 | 8.3 |
|  | Not caesarean | 4,539 | 80 | 19 | 99 | 17.6 | 4.2 | 21.8 |
| ANC visits (three categories) | 1–3 visits | 976 | 18 | 2 | 20 | 18.4 | 2.5 | 20.9 |
|  | Four-plus visits | 2,715 | 13 | 4 | 17 | 4.8 | 1.3 | 6.1 |
|  | Do ’not know/None | 236 | 3 | 1 | 4 | 14 |  | 17.3 |
| ANC visits (two categories) | 0–3 visits | 1,212 | 21 | 3 | 25 | 17.6 | 2.6 | 20.2 |
|  | Four-plus visits | 2,715 | 13 | 4 | 17 | 4.8 | 1.3 | 6.1 |
| Days iron tablets taken | <180 days | 1,936 | 26 | 2 | 28 | 13.2 | 1.3 | 14.4 |
|  | 180-plus days | 1,628 | 7 | 2 | 9 | 4.3 | 1.4 | 5.7 |
| Newborn PNC within two days | No PNC | 1,787 | 24 | 2 | 26 | 13.6 | 1.1 | 14.6 |
|  | Yes PNC | 2,140 | 10 | 5 | 15 | 4.7 | 2.3 | 7 |
| Mother PNC within two days | No PNC | 1,813 | 19 | 2 | 21 | 10.3 | 1.1 | 11.4 |
|  | Yes PNC | 2,114 | 16 | 5 | 21 | 7.4 | 2.3 | 9.7 |
