# Supplementary material for: Elucidating trends and underlying drivers of neonatal mortality stagnation in Nepal: An analytical perspective on the 2016 and 2022 Demographic and Health Surveys
Source: PLoS One. 2025 Aug 22;20(8):e0330734. doi: 10.1371/journal.pone.0330734 (PMC12373174; doi:10.1371/journal.pone.0330734)
Supplement: S2 Table — (DOCX) [file pone.0330734.s002.docx]

S2 Table : The Numbers of Births; the Numbers of Early Neonatal, Late Neonatal, and Neonatal Deaths; and the Early Neonatal, Late Neonatal, and NMRs per 1,000 Births, for all Categories of the Covariates, for the Five Years Before the 2022 NDHS.

| **Characteristics** | **Categories** | **Births** | **Early ND** | **Late ND** | **ND** | **ENMR** | **LNMR** | **NMR** |
| --- | --- | --- | --- | --- | --- | --- | --- | --- |
| National | National | 5,192 | 85 | 20 | 105 | 16.4 | 3.9 | 20.3 |
| Respondent’s language | Bhojpuri | 495 | 20 | 1 | 21 | 40.7 | 2.2 | 43 |
|  | Maithili | 1,049 | 24 | 5 | 29 | 23.1 | 4.3 | 27.4 |
|  | Nepali | 2,433 | 15 | 10 | 25 | 6.2 | 4.1 | 10.3 |
|  | Other | 1,215 | 26 | 4 | 30 | 21.1 | 3.6 | 24.7 |
| Ethnicity (three categories) | Advantaged | 2,443 | 36 | 13 | 48 | 14.6 | 5.1 | 19.7 |
|  | Disadvantaged Dalit | 1,301 | 30 | 4 | 34 | 23.3 | 2.9 | 26.3 |
|  | Disadvantaged Janajati | 1,448 | 19 | 4 | 23 | 13.2 | 2.5 | 15.8 |
| Ethnicity (two categories) | Advantaged | 1,436 | 11 | 4 | 15 | 7.8 | 2.9 | 10.7 |
|  | Disadvantaged | 3,755 | 74 | 16 | 90 | 19.7 | 4.2 | 23.9 |
| Wealth index in terciles | Poorer | 1,180 | 20 | 7 | 26 | 16.7 | 5.7 | 22.4 |
|  | Middle | 1,767 | 41 | 11 | 52 | 23.3 | 6.1 | 29.4 |
|  | Higher | 2,245 | 24 | 3 | 27 | 10.8 | 1.1 | 12 |
| Wealth index (one and two, three, four and five) | Middle | 1,079 | 22 | 2 | 24 | 20.8 | 1.9 | 22.7 |
|  | Poorer and poorest | 2,358 | 50 | 16 | 65 | 21 | 6.8 | 27.8 |
|  | Richer and richest | 1,755 | 13 | 2 | 15 | 7.5 | 1.2 | 8.7 |
| Province | Koshi | 899 | 21 | 2 | 23 | 23.5 | 2.6 | 26 |
|  | Madhesh | 1,412 | 35 | 7 | 42 | 24.9 | 4.8 | 29.7 |
|  | Bagmati | 822 | 9 | 0 | 9 | 11.2 |  | 11.2 |
|  | Gandaki | 331 | 0 | 0 | 0 |  |  |  |
|  | Lumbini | 880 | 10 | 5 | 15 | 11.6 | 5.6 | 17.2 |
|  | Karnali | 383 | 5 | 2 | 7 | 12.7 | 6.3 | 18.9 |
|  | Sudurpaschim | 466 | 5 | 4 | 8 | 10 | 7.8 | 17.8 |
| Ecological region | Hill | 1,777 | 20 | 4 | 24 | 11.1 | 2.2 | 13.2 |
|  | Mountain | 332 | 7 | 2 | 9 | 19.9 | 6.3 | 26.2 |
|  | Terai | 3,083 | 59 | 14 | 73 | 19.1 | 4.6 | 23.7 |
| Religion | Buddhist | 259 | 2 | 0 | 2 | 7.3 | 1.9 | 9.2 |
|  | Hindu | 4,344 | 67 | 18 | 84 | 15.3 | 4 | 19.4 |
|  | Muslim | 327 | 13 | 1 | 14 | 40.5 | 3.5 | 44 |
|  | Other | 262 | 3 | 1 | 4 | 12.8 | 3.3 | 16.1 |
| Type of place | Rural | 1,822 | 35 | 9 | 44 | 19.4 | 4.7 | 24.1 |
|  | Urban | 3,370 | 50 | 11 | 61 | 14.8 | 3.4 | 18.2 |
| Size of household | <six members | 2,776 | 52 | 10 | 62 | 18.8 | 3.5 | 22.2 |
|  | ≥six members | 2,416 | 33 | 10 | 44 | 13.7 | 4.3 | 18 |
| Sex of household head | Female | 1,643 | 22 | 5 | 27 | 13.5 | 2.8 | 16.3 |
|  | Male | 3,548 | 63 | 15 | 78 | 17.7 | 4.3 | 22.1 |
| Indoor air pollution | No | 2,089 | 20 | 3 | 24 | 9.8 | 1.5 | 11.3 |
|  | Yes | 3,103 | 65 | 17 | 82 | 20.9 | 5.4 | 26.3 |
| Improved water and sanitation | Improved | 3,890 | 63 | 18 | 81 | 16.2 | 4.5 | 20.7 |
|  | Not a de jure resident | 454 | 5 | 1 | 6 | 11 | 2.5 | 13.5 |
|  | Unimproved | 848 | 17 | 1 | 19 | 20.3 | 1.7 | 21.9 |
| Maternal education | Basic (grades 1–8) | 1,837 | 36 | 10 | 46 | 19.6 | 5.7 | 25.3 |
|  | No education | 1,168 | 30 | 4 | 34 | 25.6 | 3.7 | 29.3 |
|  | Secondary and above (≥grade nine) | 2,187 | 19 | 5 | 24 | 8.8 | 2.4 | 11.2 |
| Maternal age categories | 15–19 years | 267 | 6 | 2 | 8 | 22.1 | 7.1 | 29.3 |
|  | 20–24 years | 1,744 | 38 | 8 | 47 | 22.1 | 4.8 | 26.9 |
|  | 25–29 years | 1,776 | 30 | 6 | 36 | 16.9 | 3.3 | 20.2 |
|  | 30–34 years | 940 | 3 | 3 | 6 | 3.2 | 3.6 | 6.8 |
|  | 35 and above | 464 | 8 | 0 | 8 | 16.9 | 1 | 17.9 |
| Maternal age (three categories) | 15–19 years | 267 | 6 | 2 | 8 | 22.1 | 7.1 | 29.3 |
|  | 20–34 years | 4,460 | 71 | 18 | 89 | 16 | 4 | 20 |
|  | ≥35 years | 464 | 8 | 0 | 8 | 16.9 | 1 | 17.9 |
| Maternal use of tobacco | No | 4,927 | 81 | 20 | 101 | 16.5 | 4 | 20.5 |
|  | Yes | 265 | 4 | 0 | 4 | 15 | 1.7 | 16.8 |
| Maternal stature | <145 cm | 244 | 8 | 1 | 9 | 33.9 | 3.5 | 37.4 |
|  | ≥145 cm | 2,433 | 42 | 8 | 50 | 17.3 | 3.3 | 20.6 |
| Maternal anemia | Anemic | 988 | 19 | 2 | 22 | 19.4 | 2.4 | 21.8 |
|  | Not anemic | 1,671 | 33 | 7 | 40 | 19.8 | 3.9 | 23.7 |
| Mother ever drinks alcohol | Never drank alcohol | 4,791 | 78 | 20 | 98 | 16.3 | 4.2 | 20.5 |
|  | At some time | 401 | 7 | 0 | 7 | 18 |  | 18 |
| Owns mobile phone | No | 893 | 21 | 4 | 25 | 24 | 4 | 28 |
|  | Yes | 4,298 | 64 | 17 | 80 | 14.8 | 3.8 | 18.7 |
| Possesses a bank account | No | 3,099 | 71 | 14 | 85 | 22.9 | 4.6 | 27.5 |
|  | Yes | 2,093 | 14 | 6 | 20 | 6.8 | 2.8 | 9.6 |
| Internet use | Never used Internet | 1,767 | 45 | 10 | 55 | 25.4 | 5.9 | 31.3 |
|  | Used at some time | 3,424 | 40 | 10 | 50 | 11.8 | 2.8 | 14.6 |
| Empowerment: household decisions | No | 3,275 | 67 | 14 | 80 | 20.4 | 4.1 | 24.5 |
|  | Yes, can make decisions | 1,917 | 18 | 6 | 25 | 9.6 | 3.4 | 13 |
| Violence justified | Violence is not justified | 4,249 | 68 | 17 | 85 | 16 | 4.1 | 20.1 |
|  | Violence is justified | 943 | 17 | 3 | 20 | 18.3 | 2.8 | 21 |
| Empowerment: health care/family planning decisions | No | 2,104 | 41 | 14 | 55 | 19.5 | 6.4 | 25.9 |
|  | Yes | 3,088 | 44 | 6 | 51 | 14.3 | 2.1 | 16.4 |
| Newspaper/Magazine | At least once a week | 2,337 | 19 | 9 | 28 | 7.9 | 3.9 | 11.9 |
|  | Less than once a week | 2,855 | 67 | 11 | 77 | 23.3 | 3.8 | 27.1 |
| Radio/TV | Less than once a week | 2,915 | 67 | 11 | 77 | 22.9 | 3.7 | 26.6 |
|  | At least once a week | 2,277 | 19 | 9 | 28 | 8.1 | 4 | 12.2 |
| Knows about ’HMG | No | 3,624 | 62 | 12 | 73 | 17 | 3.2 | 20.2 |
|  | Yes | 1,567 | 23 | 8 | 32 | 15 | 5.4 | 20.4 |
| Husband’s education | Basic (grades 1–8) | 2,036 | 37 | 7 | 44 | 18.1 | 3.3 | 21.5 |
|  | No education/’Do not know | 695 | 21 | 5 | 26 | 30.6 | 6.9 | 37.5 |
|  | Secondary and above (≥grade nine) | 2,407 | 27 | 7 | 34 | 11.2 | 2.9 | 14.1 |
| Husband’s occupation (four categories) | Agriculture | 752 | 8 | 1 | 9 | 10.7 | 1.8 | 12.5 |
|  | Manual (skilled/unskilled) | 2,673 | 60 | 10 | 69 | 22.3 | 3.7 | 25.9 |
|  | Not working | 131 | 1 | 0 | 1 | 4.7 |  | 4.7 |
|  | Sales, clerical, other | 1,582 | 17 | 7 | 24 | 10.7 | 4.7 | 15.4 |
| Birthweight taken | Not taken | 604 | 23 | 5 | 27 | 37.3 | 7.6 | 44.9 |
|  | Yes, taken | 2,386 | 19 | 10 | 29 | 8.1 | 4.1 | 12.3 |
| Sex of child | Female | 2,458 | 31 | 9 | 40 | 12.7 | 3.6 | 16.3 |
|  | Male | 2,733 | 54 | 11 | 65 | 19.7 | 4.1 | 23.8 |
| Birthweight | Large (≥3,500 g) | 651 | 3 | 1 | 4 | 4.7 | 2.2 | 6.9 |
|  | Normal (2,500–3,500 g) | 1,455 | 14 | 4 | 18 | 9.8 | 2.5 | 12.4 |
|  | Not weighed or do not know | 604 | 23 | 5 | 27 | 37.3 | 7.6 | 44.9 |
|  | Small (<2,500 g) | 279 | 2 | 5 | 7 | 7.4 | 16.9 | 24.2 |
| Perceived birthweight | Very large | 61 | 1 | 0 | 1 | 21.2 |  | 21.2 |
|  | Larger than average | 318 | 1 | 2 | 4 | 4 | 7.2 | 11.2 |
|  | Average | 2,173 | 32 | 9 | 42 | 14.9 | 4.2 | 19.1 |
|  | Smaller than average | 299 | 2 | 1 | 4 | 7.7 | 4.8 | 12.5 |
|  | Very small | 133 | 3 | 2 | 5 | 25.4 | 12.1 | 37.5 |
|  | Do not know | 5 | 1 | 0 | 1 | 246.1 |  | 246.1 |
| Birth order | First born | 2,116 | 42 | 10 | 52 | 19.7 | 4.8 | 24.6 |
|  | 2–4 | 2,855 | 37 | 9 | 47 | 13 | 3.3 | 16.3 |
|  | Five or more | 220 | 6 | 0 | 7 | 28.1 | 2.1 | 30.2 |
| Mother’s parity | Primigravida | 2,938 | 58 | 13 | 71 | 19.6 | 4.5 | 24 |
|  | Multigravida | 2,254 | 28 | 7 | 35 | 12.3 | 3.1 | 15.3 |
| Preceding birth interval | >two years | 2,369 | 20 | 7 | 27 | 8.4 | 3.1 | 11.5 |
|  | First birth | 2,116 | 42 | 10 | 52 | 19.7 | 4.8 | 24.6 |
|  | ≤two years | 692 | 24 | 3 | 26 | 34.1 | 3.7 | 37.7 |
| Twin birth | No | 5,126 | 84 | 19 | 103 | 16.5 | 3.6 | 20.1 |
|  | Yes | 33 | 0 | 0 | 0 | 10.7 |  | 10.7 |
| Wanted last birth | Wanted then | 2,765 | 59 | 15 | 73 | 21.2 | 5.2 | 26.5 |
|  | Wanted later | 641 | 8 | 1 | 8 | 11.9 | 1.3 | 13.2 |
|  | Wanted no more | 272 | 4 | 3 | 8 | 16.5 | 12 | 28.6 |
| Time to health facility | <=30 minutes | 4,494 | 69 | 17 | 86 | 15.4 | 3.7 | 19.1 |
|  | >30 minutes | 697 | 16 | 3 | 19 | 22.8 | 4.7 | 27.5 |
| Birth attendants | Delivery without SBA | 623 | 17 | 6 | 22 | 27.2 | 8.9 | 36.1 |
|  | Delivery with SBA | 2,367 | 25 | 9 | 34 | 10.6 | 3.7 | 14.3 |
| Place of delivery | Home delivery | 598 | 16 | 6 | 21 | 26.4 | 9.5 | 35.9 |
|  | Public health facility | 1,812 | 18 | 8 | 26 | 9.8 | 4.4 | 14.1 |
|  | Private health facility | 580 | 8 | 1 | 9 | 14.6 | 1.4 | 16.1 |
| C-section past years | Caesarean | 559 | 4 | 1 | 5 | 6.7 | 2.6 | 9.3 |
|  | Not caesarean | 2,431 | 38 | 13 | 51 | 15.7 | 5.3 | 21.1 |
| ANC visits (three categories) | 1–3 visits | 443 | 9 | 2 | 11 | 21.4 | 4.3 | 25.7 |
|  | Four-plus visits | 2,251 | 19 | 6 | 25 | 8.6 | 2.5 | 11.1 |
|  | Do not know/None | 69 | 0 | 0 | 0 | 7.1 |  | 7.1 |
| ANC visits (two categories) | 0–3 visits | 511 | 10 | 2 | 12 | 19.5 | 3.8 | 23.3 |
|  | Four-plus visits | 2,252 | 19 | 6 | 25 | 8.6 | 2.5 | 11.1 |
| Days iron tablets taken | <180 days | 862 | 13 | 4 | 17 | 14.6 | 4.9 | 19.4 |
|  | 180-plus days | 1,805 | 16 | 3 | 20 | 9 | 1.9 | 10.9 |
| Newborn PNC within two days | No PNC | 838 | 18 | 4 | 22 | 21.7 | 4.6 | 26.3 |
|  | Yes PNC | 1,925 | 11 | 4 | 15 | 5.8 | 1.9 | 7.7 |
| Mother PNC within two days | No PNC | 849 | 14 | 3 | 16 | 16.2 | 3.2 | 19.4 |
|  | Yes PNC | 1,914 | 16 | 5 | 20 | 8.1 | 2.5 | 10.6 |
