# Supplementary material for: Elucidating trends and underlying drivers of neonatal mortality stagnation in Nepal: An analytical perspective on the 2016 and 2022 Demographic and Health Surveys
Source: PLoS One. 2025 Aug 22;20(8):e0330734. doi: 10.1371/journal.pone.0330734 (PMC12373174; doi:10.1371/journal.pone.0330734)
Supplement: S3 Table — (DOCX) [file pone.0330734.s003.docx]

S3 Table : The Early Neonatal Rates in the 2016 and 2022 NDHSs, and the Difference Between Them with Confidence Intervals for the Rates and the Difference and P-values for the Difference.

|  |  | **2016 with 95% confidence interval** | | | **2022 with 95% confidence interval** | | | **Difference with 95% confidence interval** | | |  |
| --- | --- | --- | --- | --- | --- | --- | --- | --- | --- | --- | --- |
| **Characteristics** | **Categories** | **ENMR** | **LB** | **UB** | **ENMR** | **LB** | **UB** | **ENMR** | **LB** | **UB** | **P-value for change** |
| National | National | 16.6 | 13.1 | 21 | 16.4 | 12.7 | 21.2 | -0.2 | -5.9 | 5.6 | 0.954 |
| Respondent’s language | Bhojpuri | 17.6 | 8.8 | 35 | 40.7 | 26.4 | 62.4 | 23.1 | 2.2 | 44.1 | 0.03 |
|  | Maithili | 16.9 | 10.6 | 26.8 | 23.1 | 13.5 | 39.1 | 6.2 | -8.2 | 20.5 | 0.401 |
|  | Nepali | 14.7 | 10.4 | 20.9 | 6.2 | 3.9 | 9.8 | -8.5 | -14.4 | -2.6 | 0.005 |
|  | Other | 18.8 | 11.6 | 30.2 | 21.1 | 13.3 | 33.5 | 2.4 | -10.9 | 15.6 | 0.727 |
| Ethnicity (three categories) | Advantaged | 14.4 | 10.4 | 19.9 | 14.6 | 10.2 | 20.8 | 0.2 | -6.8 | 7.2 | 0.951 |
|  | Disadvantaged Dalit | 21.1 | 12.8 | 34.6 | 23.3 | 14.5 | 37.5 | 2.3 | -13 | 17.5 | 0.77 |
|  | Disadvantaged Janajati | 17.3 | 10.8 | 27.4 | 13.2 | 7.8 | 22.4 | -4 | -14.7 | 6.6 | 0.456 |
| Ethnicity (two categories) | Advantaged | 15 | 10.3 | 21.9 | 7.8 | 4.6 | 13.2 | -7.2 | -14.2 | -0.2 | 0.043 |
|  | Disadvantaged | 17.3 | 13.0 | 23.1 | 19.7 | 14.9 | 26 | 2.4 | -5 | 9.8 | 0.526 |
| Wealth index in terciles | Poorer | 19.3 | 13.2 | 28 | 16.7 | 11 | 25.3 | -2.6 | -12.6 | 7.5 | 0.616 |
|  | Middle | 18.2 | 12.4 | 26.7 | 23.3 | 16.9 | 32 | 5.1 | -5.1 | 15.3 | 0.33 |
|  | Higher | 13 | 8.3 | 20.2 | 10.8 | 6.5 | 18.1 | -2.1 | -10.1 | 5.9 | 0.603 |
| Wealth index (one and two, three, four and five) | Middle | 13.4 | 7.2 | 25.1 | 20.8 | 12.2 | 35 | 7.3 | -6.4 | 21.1 | 0.297 |
|  | Poorer and poorest | 22.1 | 16.2 | 30.1 | 21 | 16 | 27.5 | -1.1 | -10 | 7.8 | 0.81 |
|  | Richer and richest | 11.9 | 7.3 | 19.2 | 7.5 | 3.9 | 14.3 | -4.3 | -11.8 | 3.1 | 0.254 |
| Province | Koshi | 17.1 | 8.3 | 34.9 | 23.5 | 13.2 | 41.5 | 6.4 | -11.7 | 24.5 | 0.486 |
|  | Madhesh | 17.3 | 11.2 | 26.6 | 24.9 | 17 | 36.4 | 7.6 | -4.4 | 19.6 | 0.215 |
|  | Bagmati | 10.9 | 5.1 | 23.1 | 11.2 | 4.8 | 26 | 0.3 | -12.2 | 12.7 | 0.967 |
|  | Gandaki |  |  |  |  |  |  | 0 | 0 | 0 |  |
|  | Lumbini | 14.1 | 7.3 | 26.9 | 11.6 | 5.5 | 24.4 | -2.5 | -15 | 10 | 0.691 |
|  | Karnali | 26.4 | 16.4 | 42.3 | 12.7 | 7.6 | 20.9 | -13.8 | -27.7 | 0.2 | 0.053 |
|  | Sudurpaschim | 26.7 | 15 | 47.2 | 10 | 4.4 | 22.5 | -16.7 | -33.9 | 0.5 | 0.057 |
| Ecological region | Hill | 14.5 | 9.2 | 22.7 | 11.1 | 7.3 | 16.7 | -3.5 | -11.4 | 4.5 | 0.395 |
|  | Mountain | 28.5 | 16.6 | 48.5 | 19.9 | 8 | 48.8 | -8.6 | -31.8 | 14.6 | 0.467 |
|  | Terai | 16.4 | 11.9 | 22.6 | 19.1 | 13.7 | 26.6 | 2.7 | -5.5 | 10.9 | 0.525 |
| Religion | Buddhist | 7.3 | 1 | 48.9 | 7.3 | 1.6 | 32.2 | 0.1 | -17.6 | 17.7 | 0.995 |
|  | Hindu | 17.8 | 13.9 | 22.8 | 15.3 | 11.8 | 20 | -2.5 | -8.5 | 3.6 | 0.425 |
|  | Muslim | 15.2 | 5.3 | 42.5 | 40.5 | 18.3 | 87.4 | 25.3 | -9.6 | 60.2 | 0.155 |
| Type of place | Rural | 21.5 | 15.7 | 29.4 | 19.4 | 14.6 | 25.7 | -2.1 | -10.8 | 6.6 | 0.633 |
|  | Urban | 12.4 | 8.6 | 17.7 | 14.8 | 10 | 21.8 | 2.4 | -4.9 | 9.7 | 0.518 |
| Size of household | <six members | 18 | 12.7 | 25.4 | 18.8 | 13.9 | 25.3 | 0.8 | -7.6 | 9.2 | 0.853 |
|  | ≥six members | 15.3 | 11.2 | 21 | 13.7 | 8.7 | 21.4 | -1.6 | -9.4 | 6.2 | 0.681 |
| Sex of household head | Female | 15.7 | 9.5 | 25.8 | 13.5 | 8.6 | 21.2 | -2.2 | -12.1 | 7.8 | 0.669 |
|  | Male | 16.9 | 13 | 22.1 | 17.7 | 13.2 | 23.7 | 0.8 | -6.1 | 7.7 | 0.819 |
| Indoor air pollution | No | 11.8 | 6.7 | 20.5 | 9.8 | 5.8 | 16.5 | -2 | -10.3 | 6.3 | 0.639 |
|  | Yes | 18.7 | 14.5 | 24.2 | 20.9 | 15.7 | 27.6 | 2.1 | -5.4 | 9.7 | 0.58 |
| Improved water and sanitation | Improved | 16.4 | 12.2 | 22.1 | 16.2 | 11.9 | 22.1 | -0.2 | -7.2 | 6.8 | 0.956 |
|  | Not a de jure resident | 26.4 | 12.6 | 54.5 | 11 | 4 | 30.1 | -15.4 | -37.7 | 6.9 | 0.177 |
|  | Unimproved | 14.3 | 8.8 | 23.1 | 20.3 | 11.5 | 35.4 | 6 | -7.3 | 19.3 | 0.375 |
| Maternal education | Basic (grade 1–8) | 22.1 | 13.6 | 35.6 | 19.6 | 13.4 | 28.5 | -2.5 | -15.3 | 10.4 | 0.708 |
|  | No education | 19.6 | 13.8 | 27.9 | 25.6 | 16.1 | 40.5 | 6 | -7.7 | 19.7 | 0.389 |
|  | Secondary and above (≥grade nine) | 11.8 | 7.5 | 18.4 | 8.8 | 5.5 | 14.1 | -3 | -9.7 | 3.7 | 0.38 |
| Maternal age (five categories) | 15–19 years | 29 | 14 | 59.2 | 22.1 | 9.8 | 49.3 | -6.9 | -34.4 | 20.7 | 0.625 |
|  | 20–24 years | 19.7 | 13.3 | 29.2 | 22.1 | 15.7 | 31 | 2.4 | -8.4 | 13.2 | 0.667 |
|  | 25–29 years | 13.3 | 8.7 | 20.5 | 16.9 | 11 | 25.8 | 3.5 | -5.7 | 12.7 | 0.454 |
|  | 30–34 years | 13.4 | 6.6 | 26.8 | 3.2 | 1 | 9.8 | -10.2 | -20.2 | -0.2 | 0.045 |
|  | 35 and above | 13.4 | 5.4 | 33 | 16.9 | 7 | 39.8 | 3.4 | -15.6 | 22.4 | 0.724 |
| Maternal age (three categories) | 15–19 years | 29 | 14 | 59.2 | 22.1 | 9.8 | 49.3 | -6.9 | -34.4 | 20.7 | 0.625 |
|  | 20–34 years | 15.8 | 12.1 | 20.7 | 16 | 12 | 21.3 | 0.2 | -6 | 6.4 | 0.951 |
|  | ≥35 years | 13.4 | 5.4 | 33 | 16.9 | 7 | 39.8 | 3.4 | -15.6 | 22.4 | 0.724 |
| Maternal use of tobacco | No | 16.8 | 13.2 | 21.5 | 16.5 | 12.6 | 21.5 | -0.3 | -6.4 | 5.7 | 0.91 |
|  | Yes | 12.4 | 5.7 | 26.7 | 15 | 7 | 31.9 | 2.6 | -12.2 | 17.4 | 0.728 |
| Maternal stature | <145 cm | 14.4 | 5.5 | 37.3 | 33.9 | 16.8 | 67.2 | 19.4 | -7.8 | 46.7 | 0.161 |
|  | ≥145 cm | 19.4 | 14.1 | 26.7 | 17.3 | 11.7 | 25.5 | -2.1 | -11.2 | 7 | 0.654 |
| Maternal anemia | Anemic | 24.4 | 16.4 | 36.2 | 19.4 | 12.3 | 30.5 | -5 | -18.1 | 8.1 | 0.454 |
|  | Not anemic | 14 | 8.6 | 22.6 | 19.8 | 12.7 | 30.7 | 5.8 | -5.2 | 16.8 | 0.303 |
| Owns mobile phone | No | 19.6 | 12.5 | 30.6 | 24 | 13.7 | 41.9 | 4.4 | -11.6 | 20.4 | 0.592 |
|  | Yes | 15.7 | 11.8 | 20.8 | 14.8 | 11.2 | 19.7 | -0.8 | -7 | 5.3 | 0.789 |
| Possesses a (bank account) | No | 17.2 | 12.8 | 23.2 | 22.9 | 17.2 | 30.5 | 5.6 | -2.7 | 14 | 0.184 |
|  | Yes | 15.3 | 10.2 | 23 | 6.8 | 4.2 | 11 | -8.6 | -15.6 | -1.5 | 0.017 |
| Internet use | Never used Internet | 17.8 | 13.7 | 23.1 | 25.4 | 18.6 | 34.7 | 7.6 | -1.6 | 16.8 | 0.104 |
|  | Used at some time | 11.2 | 6 | 20.8 | 11.8 | 7.9 | 17.4 | 0.6 | -7.8 | 8.9 | 0.895 |
| Empowerment: household decisions | No | 18.1 | 13.7 | 23.9 | 20.4 | 15.4 | 26.8 | 2.3 | -5.3 | 9.8 | 0.553 |
|  | Yes, can make decisions | 14.2 | 9.2 | 21.7 | 9.6 | 5.9 | 15.6 | -4.5 | -12.2 | 3.1 | 0.247 |
| Violence justified | Violence is not justified | 16.1 | 12.3 | 21.2 | 16 | 12.3 | 20.8 | -0.2 | -6.2 | 5.9 | 0.961 |
|  | Violence is justified | 17.6 | 11.2 | 27.7 | 18.3 | 9.7 | 34.3 | 0.6 | -13.4 | 14.7 | 0.931 |
| Empowerment: health care/family planning decisions | No | 19.3 | 15.1 | 24.6 | 19.5 | 13.8 | 27.4 | 0.2 | -8 | 8.4 | 0.967 |
|  | Yes | 5.1 | 2 | 12.7 | 14.3 | 10.4 | 19.6 | 9.3 | 2.8 | 15.8 | 0.005 |
| Newspaper/Magazine | At least once a week | 15 | 10.9 | 20.7 | 7.9 | 4.9 | 12.8 | -7.1 | -13.2 | -1 | 0.023 |
|  | Less than once a week | 18.3 | 13.1 | 25.6 | 23.3 | 17.7 | 30.8 | 5 | -3.9 | 13.9 | 0.269 |
| Radio/TV | Less than once a week | 18.2 | 13 | 25.4 | 22.9 | 17.3 | 30.2 | 4.6 | -4.2 | 13.4 | 0.303 |
|  | At least once a week | 15.1 | 10.9 | 20.8 | 8.1 | 5 | 13.1 | -7 | -13.2 | -0.8 | 0.028 |
| Knows about HMG | No | 17 | 12.8 | 22.6 | 17 | 12.3 | 23.5 | 0 | -7.4 | 7.3 | 0.993 |
|  | Yes | 15.6 | 10.5 | 23.1 | 15 | 10.2 | 22 | -0.6 | -9.1 | 7.8 | 0.885 |
| Husband’s education | Basic (grades 1–8) | 27.3 | 18.3 | 40.5 | 18.1 | 12.6 | 26.1 | -9.1 | -21.8 | 3.5 | 0.157 |
|  | No education/Do not know | 14.6 | 7.6 | 28 | 30.6 | 18.3 | 50.9 | 16 | -2.3 | 34.3 | 0.087 |
|  | Secondary and above (≥grade nine) | 13.3 | 9.4 | 18.7 | 11.2 | 7.1 | 17.7 | -2.1 | -9 | 4.8 | 0.548 |
| Husband’s occupation (four categories) | Agriculture | 23.9 | 16.1 | 35.3 | 10.7 | 5.9 | 19.3 | -13.3 | -24.6 | -2 | 0.021 |
|  | Manual (skilled/unskilled) | 17.6 | 12 | 25.8 | 22.3 | 16.3 | 30.3 | 4.6 | -5 | 14.3 | 0.345 |
|  | Sales, clerical, other | 13.5 | 9 | 20.3 | 10.7 | 6.5 | 17.7 | -2.8 | -10.5 | 4.9 | 0.48 |
| Birthweight taken | Not taken | 30 | 22.4 | 40.1 | 37.3 | 21.1 | 65.2 | 7.3 | -15.5 | 30.1 | 0.53 |
|  | Yes, taken | 7.8 | 5.0 | 12.1 | 8.1 | 4.8 | 13.7 | 0.4 | -5.1 | 5.8 | 0.893 |
| Sex of child | Female | 14.3 | 10 | 20.5 | 12.7 | 8.5 | 19.2 | -1.6 | -8.9 | 5.7 | 0.673 |
|  | Male | 18.6 | 13.6 | 25.5 | 19.7 | 14.3 | 27.1 | 1.1 | -7.5 | 9.7 | 0.809 |
| Birthweight | Large (≥3,500 g) | 7.9 | 3.8 | 16.5 | 4.7 | 1.4 | 15.6 | -3.2 | -11.3 | 4.9 | 0.435 |
|  | Normal (2,500–3,500 g) | 4.2 | 2 | 8.8 | 9.8 | 5.2 | 18.6 | 5.6 | -1.4 | 12.7 | 0.114 |
|  | Not weighed or do not know | 30 | 22.4 | 40.1 | 37.3 | 21.1 | 65.2 | 7.3 | -15.5 | 30.1 | 0.530 |
|  | Small (<2,500 g) | 22.2 | 10.6 | 46.1 | 7.4 | 2.9 | 18.8 | -14.9 | -32.6 | 2.9 | 0.10 |
| Perceived birthweight | Very large | 26.5 | 10.5 | 65.2 | 21.2 | 4.9 | 86.9 | -5.3 | -44.1 | 33.5 | 0.789 |
|  | Larger than average | 23.7 | 13.5 | 41.3 | 4 | 1 | 15.4 | -19.8 | -34 | -5.5 | 0.007 |
|  | Average | 10.8 | 7.6 | 15.3 | 14.9 | 9.7 | 22.9 | 4.2 | -3.2 | 11.6 | 0.268 |
|  | Smaller than average | 23.5 | 12.8 | 42.6 | 7.7 | 2.5 | 23.5 | -15.7 | -32.2 | 0.8 | 0.062 |
|  | Very small | 32.8 | 16 | 65.9 | 25.4 | 9.4 | 66.7 | -7.4 | -41.3 | 26.5 | 0.669 |
| Birth order | First born | 19.9 | 14.1 | 28 | 19.7 | 14.2 | 27.4 | -0.2 | -9.6 | 9.2 | 0.967 |
|  | 2–4 | 13.2 | 9.3 | 18.8 | 13 | 8.6 | 19.6 | -0.2 | -7.3 | 6.9 | 0.953 |
|  | Five or more | 21.4 | 11.2 | 40.6 | 28.1 | 12.8 | 60.8 | 6.7 | -19.2 | 32.6 | 0.612 |
| Mother’s parity | Primigravida | 19.6 | 14.3 | 26.9 | 19.6 | 14.4 | 26.6 | 0 | -8.7 | 8.6 | 0.995 |
|  | Multigravida | 13.2 | 9.2 | 18.9 | 12.3 | 7.8 | 19.1 | -0.9 | -8.1 | 6.3 | 0.804 |
| Preceding birth interval | >two years | 10.8 | 7.1 | 16.4 | 8.4 | 5.2 | 13.5 | -2.5 | -8.5 | 3.6 | 0.426 |
|  | First birth | 19.9 | 14.1 | 28 | 19.7 | 14.2 | 27.4 | -0.2 | -9.6 | 9.2 | 0.967 |
|  | ≤two years | 23.7 | 13.5 | 41 | 34.1 | 19.6 | 58.7 | 10.4 | -12.4 | 33.2 | 0.371 |
| Twin birth | No | 15.7 | 12.3 | 20 | 16.5 | 12.7 | 21.3 | 0.8 | -4.9 | 6.5 | 0.784 |
|  | Yes | 74.3 | 21.4 | 227.5 | 10.7 | 1.4 | 76.2 | -63.6 | -152.8 | 25.6 | 0.162 |
| Wanted last birth | Wanted then | 17 | 12.9 | 22.3 | 21.2 | 15.5 | 29 | 4.3 | -3.8 | 12.4 | 0.303 |
|  | Wanted later | 15.6 | 8 | 30.3 | 11.9 | 5.3 | 26.2 | -3.7 | -17.7 | 10.3 | 0.604 |
|  | Wanted no more | 16.4 | 7.7 | 34.6 | 16.5 | 5.9 | 45.4 | 0.1 | -20.8 | 20.9 | 0.994 |
| Time to health facility | <=30 minutes | 1.8 | 0.4 | 7.3 | 15.4 | 11.7 | 20.3 | 13.6 | 8.7 | 18.6 | 0 |
|  | >30 minutes | 9.2 | 5 | 17 | 22.8 | 12.6 | 40.9 | 13.6 | -0.9 | 28.2 | 0.067 |
| Birth attendants | Delivery without SBA | 22.6 | 16.3 | 31.2 | 27.2 | 13.4 | 54.4 | 4.6 | -15.8 | 25 | 0.659 |
|  | Delivery with SBA | 11.9 | 8.4 | 17 | 10.6 | 6.8 | 16.4 | -1.4 | -7.6 | 4.9 | 0.671 |
| Place of delivery | Home delivery | 22.3 | 16.1 | 30.9 | 26.4 | 12.5 | 55.3 | 4.1 | -16.9 | 25.1 | 0.699 |
|  | Public health facility | 11.1 | 7.2 | 17.1 | 9.8 | 5.6 | 17 | -1.3 | -8.6 | 5.9 | 0.719 |
|  | Private health facility | 15.5 | 8.6 | 27.6 | 14.6 | 7.7 | 27.8 | -0.8 | -13.9 | 12.2 | 0.899 |
| C-section past years | Caesarean | 4.6 | 1.1 | 18.4 | 6.7 | 2.1 | 21.3 | 2.1 | -8.0 | 12.2 | 0.683 |
|  | Not caesarean | 17.6 | 13.8 | 22.4 | 15.7 | 10.5 | 23.6 | -1.9 | -9.5 | 5.8 | 0.634 |
| ANC visits (three categories) | 1–3 visits | 18.4 | 10.8 | 31.2 | 21.4 | 10.5 | 43.2 | 2.9 | -15.1 | 20.9 | 0.750 |
|  | Four-plus visits | 4.8 | 2.7 | 8.6 | 8.6 | 5.1 | 14.4 | 3.8 | -1.5 | 9 | 0.163 |
|  | Do not know/None | 14 | 4.6 | 42.2 | 7.1 | 1 | 49.2 | -6.9 | -27.7 | 13.9 | 0.516 |
| ANC visits (two categories) | 0–3 visits | 17.6 | 11 | 28 | 19.5 | 9.8 | 38.5 | 1.9 | -13.7 | 17.6 | 0.81 |
|  | Four-plus visits | 4.8 | 2.7 | 8.6 | 8.6 | 5.1 | 14.4 | 3.8 | -1.5 | 9 | 0.163 |
| Days iron tablets taken | <180 days | 13.2 | 8.3 | 20.8 | 14.6 | 7.8 | 27 | 1.4 | -9.5 | 12.3 | 0.802 |
|  | 180-plus days | 4.3 | 2 | 9.5 | 9 | 5 | 15.9 | 4.6 | -1.5 | 10.8 | 0.14 |
| Newborn PNC within two days | No PNC | 13.6 | 8.7 | 21.1 | 21.7 | 12.4 | 37.6 | 8.1 | -5.3 | 21.5 | 0.238 |
|  | Yes PNC | 4.7 | 2.5 | 8.8 | 5.8 | 2.9 | 11.2 | 1 | -3.8 | 5.9 | 0.675 |
| Mother PNC within two days | No PNC | 10.3 | 6.4 | 16.7 | 16.2 | 8.6 | 30.1 | 5.9 | -5.4 | 17.1 | 0.306 |
|  | Yes PNC | 7.4 | 4.2 | 13 | 8.1 | 4.5 | 14.6 | 0.7 | -5.6 | 7 | 0.83 |

Note: LB indicates lower bound and UB indicates upper bound.
