# Supplementary material for: Elucidating trends and underlying drivers of neonatal mortality stagnation in Nepal: An analytical perspective on the 2016 and 2022 Demographic and Health Surveys
Source: PLoS One. 2025 Aug 22;20(8):e0330734. doi: 10.1371/journal.pone.0330734 (PMC12373174; doi:10.1371/journal.pone.0330734)
Supplement: S4 Table — (DOCX) [file pone.0330734.s004.docx]

S4 Table: The Late Neonatal Rates in the 2016 and 2022 NDHSs and the Difference Between Them, with Confidence Intervals for the Rates and the Difference and P-values for the Difference.

| **Characteristics** | **Categories** | **2016 with 95%**  **confidence interval** | | | **2022 with 95%**  **confidence interval** | | | **Difference with 95%**  **confidence interval** | | | **P-value for difference** |
| --- | --- | --- | --- | --- | --- | --- | --- | --- | --- | --- | --- |
|  |  | **LNMR** | **LB** | **UB** | **LNMR** | **LB** | **UB** | **LNMR** | **LB** | **UB** |  |
| National | National | 4.3 | 2.6 | 7.1 | 3.9 | 2.5 | 5.9 | -0.5 | -3.2 | 2.3 | 0.737 |
| Respondent’s language | Bhojpuri | 8.2 | 2.4 | 27.5 | 2.2 | 0.6 | 8.8 | -6 | -16.2 | 4.2 | 0.25 |
|  | Maithili | 6.5 | 2.3 | 18.4 | 4.3 | 1.4 | 13.3 | -2.2 | -10.4 | 6.1 | 0.61 |
|  | Nepali | 2.6 | 1.1 | 6.4 | 4.1 | 2.3 | 7.3 | 1.5 | -1.8 | 4.8 | 0.379 |
|  | Other | 3.5 | 1.4 | 8.6 | 3.6 | 1.5 | 8.7 | 0.1 | -4.3 | 4.6 | 0.953 |
| Ethnicity (three categories) | Advantaged | 4 | 1.9 | 8.5 | 5.1 | 2.9 | 9.1 | 1.1 | -3.1 | 5.3 | 0.609 |
|  | Disadvantaged Dalit | 5.9 | 2.5 | 14.1 | 2.9 | 1.3 | 6.8 | -3 | -8.7 | 2.7 | 0.302 |
|  | Disadvantaged Janajati | 3.6 | 1.2 | 11 | 2.5 | 0.9 | 7.2 | -1.1 | -5.9 | 3.7 | 0.654 |
| Ethnicity (two categories) | Advantaged | 3.3 | 1.1 | 9.9 | 2.9 | 1.4 | 6 | -0.4 | -4.6 | 3.8 | 0.843 |
|  | Disadvantaged | 4.8 | 2.7 | 8.4 | 4.2 | 2.5 | 7.1 | -0.6 | -4 | 2.9 | 0.749 |
| Wealth index in terciles | Poorer | 4.8 | 2.2 | 10.4 | 5.7 | 2.8 | 11.3 | 0.9 | -4.5 | 6.3 | 0.752 |
|  | Middle | 3.2 | 1.1 | 9.1 | 6.1 | 3.3 | 11.4 | 2.9 | -2.2 | 8 | 0.26 |
|  | Higher | 5 | 2.2 | 11.5 | 1.1 | 0.3 | 3.9 | -3.9 | -8.3 | 0.5 | 0.081 |
| Wealth index (one and two, three, four and five) | Middle | 2.4 | 0.7 | 8.4 | 1.9 | 0.5 | 6.7 | -0.4 | -4.3 | 3.4 | 0.82 |
|  | Poorer and poorest | 5.2 | 2.6 | 10.2 | 6.8 | 4.2 | 10.9 | 1.6 | -3.2 | 6.3 | 0.525 |
|  | Richer and richest | 4.5 | 2 | 10.1 | 1.2 | 0.3 | 5.1 | -3.3 | -7.4 | 0.7 | 0.104 |
| Province | Koshi | 5.8 | 1.7 | 19.7 | 2.6 | 0.8 | 8.2 | -3.2 | -10.9 | 4.4 | 0.412 |
|  | Madhesh | 7 | 3.1 | 15.7 | 4.8 | 2 | 11.2 | -2.2 | -9.1 | 4.7 | 0.532 |
|  | Bagmati |  |  |  |  |  |  |  |  |  |  |
|  | Gandaki |  |  |  |  |  |  |  |  |  |  |
|  | Lumbini | 4.3 | 1.4 | 13.2 | 5.6 | 2.1 | 14.8 | 1.3 | -5.9 | 8.6 | 0.718 |
|  | Karnali | 4.9 | 1.8 | 13.5 | 6.3 | 2.9 | 13.5 | 1.4 | -5.5 | 8.2 | 0.693 |
|  | Sudurpaschim | 3.2 | 0.8 | 13 | 7.8 | 3.4 | 17.5 | 4.6 | -3.1 | 12.3 | 0.239 |
| Ecological region | Hill | 1.8 | 0.6 | 5.2 | 2.2 | 1.1 | 4.3 | 0.4 | -2.1 | 2.8 | 0.776 |
|  | Mountain | 7.9 | 2.8 | 22.6 | 6.3 | 2.2 | 17.9 | -1.7 | -12.1 | 8.7 | 0.751 |
|  | Terai | 5.6 | 2.9 | 10.5 | 4.6 | 2.6 | 8 | -1 | -5.4 | 3.4 | 0.655 |
| Religion | Buddhist |  |  |  | 1.9 | 0.3 | 13.7 |  |  |  | 0 |
|  | Hindu | 4.7 | 2.8 | 7.9 | 4 | 2.5 | 6.4 | -0.7 | -3.8 | 2.4 | 0.664 |
|  | Muslim | 4.3 | 0.6 | 29 | 3.5 | 0.5 | 25.6 | -0.7 | -11.4 | 9.9 | 0.893 |
| Type of place | Rural | 4.8 | 2.3 | 10 | 4.7 | 2.6 | 8.5 | -0.1 | -4.6 | 4.4 | 0.978 |
|  | Urban | 4 | 2 | 7.8 | 3.4 | 1.8 | 6.3 | -0.6 | -4 | 2.9 | 0.751 |
| Size of household | <six members | 3.3 | 1.4 | 7.9 | 3.5 | 1.8 | 6.6 | 0.2 | -3.5 | 3.8 | 0.92 |
|  | ≥six members | 5.3 | 2.9 | 9.6 | 4.3 | 2.4 | 7.8 | -1 | -5 | 3.1 | 0.646 |
| Sex of household head | Female | 1.6 | 0.4 | 6.4 | 2.8 | 1.2 | 6.5 | 1.2 | -2.1 | 4.5 | 0.47 |
|  | Male | 5.4 | 3.3 | 9 | 4.3 | 2.6 | 7.2 | -1.1 | -4.6 | 2.4 | 0.544 |
| Indoor air pollution | No | 5.6 | 2.3 | 13.4 | 1.5 | 0.5 | 4.9 | -4.1 | -9.3 | 1.1 | 0.123 |
|  | Yes | 3.8 | 2 | 6.9 | 5.4 | 3.4 | 8.6 | 1.7 | -1.7 | 5.1 | 0.332 |
| Improved water and sanitation | Improved | 3.2 | 1.7 | 6 | 4.5 | 2.8 | 7.2 | 1.3 | -1.6 | 4.2 | 0.379 |
|  | Not a de jure resident | 11.6 | 3.9 | 34.2 | 2.5 | 0.3 | 17.4 | -9.2 | -22.7 | 4.4 | 0.184 |
|  | Unimproved | 5 | 1.9 | 13.2 | 1.7 | 0.4 | 6.9 | -3.4 | -8.8 | 2 | 0.221 |
| Maternal education | Basic (grades 1–8) | 1.3 | 0.2 | 8.9 | 5.7 | 3.1 | 10.3 | 4.4 | 0.2 | 8.6 | 0.039 |
|  | No education | 6.1 | 3 | 12.4 | 3.7 | 1.2 | 11.1 | -2.4 | -8.3 | 3.5 | 0.428 |
|  | Secondary and above (≥grade nine) | 4.3 | 2.2 | 8.6 | 2.4 | 1.2 | 4.9 | -1.9 | -5.4 | 1.5 | 0.268 |
| Maternal age (five categories) | 15–19 years | 4.2 | 0.6 | 27.9 | 7.1 | 2.1 | 23.6 | 3 | -8.7 | 14.7 | 0.621 |
|  | 20–24 years | 8 | 4.2 | 14.9 | 4.8 | 2.6 | 9 | -3.2 | -9 | 2.7 | 0.289 |
|  | 25–29 years | 2.7 | 1 | 7.2 | 3.3 | 1.4 | 8 | 0.7 | -3.3 | 4.6 | 0.747 |
|  | 30–34 years | 1.6 | 0.2 | 11.2 | 3.6 | 1.1 | 12.1 | 2.1 | -3.3 | 7.4 | 0.453 |
|  | 35 and above | 2.7 | 0.4 | 18.8 | 1 | 0.1 | 7 | -1.7 | -7.2 | 3.9 | 0.556 |
| Maternal age (three categories) | 15–19 years | 4.2 | 0.6 | 27.9 | 7.1 | 2.1 | 23.6 | 3 | -8.7 | 14.7 | 0.621 |
|  | 20–34 years | 4.5 | 2.6 | 7.7 | 4 | 2.5 | 6.3 | -0.5 | -3.6 | 2.5 | 0.727 |
|  | ≥35 years | 2.7 | 0.4 | 18.8 | 1 | 0.1 | 7 | -1.7 | -7.2 | 3.9 | 0.556 |
| Maternal use of tobacco | No | 4.5 | 2.7 | 7.4 | 4 | 2.6 | 6.2 | -0.5 | -3.4 | 2.4 | 0.738 |
|  | Yes | 2.1 | 0.3 | 14.6 | 1.7 | 0.2 | 12.4 | -0.4 | -5.7 | 4.9 | 0.894 |
| Maternal stature | <145 cm | 14.7 | 5.2 | 41.2 | 3.5 | 0.5 | 24.9 | -11.2 | -28 | 5.5 | 0.189 |
|  | ≥145 cm | 2.9 | 1.3 | 6.4 | 3.3 | 1.7 | 6.5 | 0.4 | -2.7 | 3.6 | 0.784 |
| Maternal anemia | Anemic | 4.6 | 1.8 | 11.6 | 2.4 | 0.9 | 6.8 | -2.1 | -7.1 | 2.8 | 0.393 |
|  | Not anemic | 3.9 | 1.3 | 11.5 | 3.9 | 1.8 | 8.5 | 0 | -5.2 | 5.3 | 0.987 |
| Owns mobile phone | No | 9.2 | 4.9 | 17 | 4 | 1.1 | 14.1 | -5.2 | -12.8 | 2.4 | 0.176 |
|  | Yes | 2.9 | 1.4 | 6 | 3.8 | 2.4 | 6.1 | 1 | -1.8 | 3.7 | 0.489 |
| Possesses a bank account | No | 5.7 | 3.4 | 9.6 | 4.6 | 2.7 | 7.7 | -1.1 | -4.9 | 2.7 | 0.573 |
|  | Yes | 1.9 | 0.6 | 6.3 | 2.8 | 1.3 | 6.1 | 0.9 | -2.2 | 4.1 | 0.568 |
| Internet use | Never used Internet | 4.8 | 2.8 | 8.1 | 5.9 | 3.1 | 11.1 | 1.2 | -3.4 | 5.7 | 0.616 |
|  | Used at some time | 2.5 | 0.6 | 10.6 | 2.8 | 1.6 | 5 | 0.3 | -3.6 | 4.3 | 0.867 |
| Empowerment: household decisions | No | 4.6 | 2.5 | 8.3 | 4.1 | 2.5 | 7 | -0.5 | -4 | 3 | 0.792 |
|  | Yes, can make decisions | 3.9 | 1.7 | 8.8 | 3.4 | 1.5 | 7.4 | -0.5 | -4.6 | 3.6 | 0.814 |
| Violence justified | Violence is not justified | 3.8 | 2.1 | 6.8 | 4.1 | 2.6 | 6.5 | 0.3 | -2.6 | 3.2 | 0.838 |
|  | Violence is justified | 5.6 | 2.4 | 13 | 2.8 | 0.8 | 9.2 | -2.9 | -8.7 | 2.9 | 0.331 |
| Empowerment: health care/family planning decisions | No | 4.1 | 2.4 | 7.2 | 6.4 | 3.7 | 11.1 | 2.3 | -1.9 | 6.5 | 0.279 |
|  | Yes | 5.1 | 1.9 | 13.8 | 2.1 | 1 | 4.4 | -3 | -8.3 | 2.3 | 0.266 |
| Newspaper/Magazine | At least once a week | 4.4 | 2.3 | 8.6 | 3.9 | 2.2 | 7.1 | -0.5 | -4.2 | 3.3 | 0.806 |
|  | Less than once a week | 4.3 | 2 | 9.1 | 3.8 | 2 | 7.1 | -0.4 | -4.5 | 3.6 | 0.828 |
| Radio/TV | Less than once a week | 4.2 | 2 | 9.1 | 3.7 | 2 | 7 | -0.5 | -4.5 | 3.5 | 0.804 |
|  | At least once a week | 4.4 | 2.3 | 8.6 | 4 | 2.2 | 7.3 | -0.4 | -4.2 | 3.4 | 0.843 |
| Knows about HMG | No | 4.6 | 2.5 | 8.6 | 3.2 | 1.8 | 5.6 | -1.4 | -4.8 | 2 | 0.417 |
|  | Yes | 3.8 | 1.6 | 8.7 | 5.4 | 2.7 | 10.7 | 1.6 | -3.2 | 6.5 | 0.514 |
| Husband’s education | Basic (grades 1–8) | 2.6 | 0.5 | 13.2 | 3.3 | 1.4 | 7.7 | 0.7 | -4.4 | 5.8 | 0.789 |
|  | No education/Do not know | 4.8 | 1.6 | 14.1 | 6.9 | 2.8 | 16.7 | 2 | -6 | 10 | 0.622 |
|  | Secondary and above (≥grade nine) | 4.9 | 2.7 | 8.9 | 2.9 | 1.6 | 5.4 | -2 | -5.4 | 1.5 | 0.263 |
| Husband’s occupation (four categories) | Agriculture | 6.7 | 2.9 | 15.2 | 1.8 | 0.4 | 8.1 | -4.9 | -11 | 1.3 | 0.12 |
|  | Manual (skilled/unskilled) | 3 | 1.1 | 8.6 | 3.7 | 1.9 | 7 | 0.6 | -3.3 | 4.6 | 0.753 |
|  | Not working |  |  |  |  |  |  |  |  |  |  |
|  | Sales, clerical, other | 5 | 2.4 | 10.1 | 4.7 | 2.4 | 9.1 | -0.3 | -5 | 4.4 | 0.909 |
| Birthweight taken | Not taken | 3.2 | 1.4 | 7.5 | 7.6 | 2.6 | 21.8 | 4.4 | -4.1 | 12.9 | 0.312 |
|  | Yes, taken | 4.8 | 2.6 | 8.8 | 4.1 | 2.2 | 7.7 | -0.7 | -4.5 | 3.2 | 0.742 |
| Sex of child | Female | 3 | 1.2 | 7.2 | 3.6 | 1.9 | 6.7 | 0.6 | -2.9 | 4 | 0.754 |
|  | Male | 5.5 | 3 | 10.3 | 4.1 | 2.3 | 7.5 | -1.4 | -5.6 | 2.8 | 0.516 |
| Birthweight | Large (≥3,500 g) | 3.1 | 0.8 | 12.4 | 2.2 | 0.3 | 15.5 | -0.9 | -7 | 5.2 | 0.779 |
|  | Normal (2,500–3,500 g) | 5.6 | 2.8 | 11.2 | 2.5 | 1 | 6.2 | -3.1 | -7.6 | 1.4 | 0.18 |
|  | Not weighed or do not know | 3.2 | 1.4 | 7.5 | 7.6 | 2.6 | 21.8 | 4.4 | -4.1 | 12.9 | 0.312 |
|  | Small (<2,500 g) | 5 | 1.1 | 22 | 16.9 | 6.5 | 43.1 | 11.9 | -5.7 | 29.5 | 0.186 |
| Perceived birthweight | Very large |  |  |  |  |  |  |  |  |  |  |
|  | Larger than average | 8.5 | 3.4 | 21.3 | 7.2 | 1.9 | 26.5 | -1.3 | -13.5 | 11 | 0.84 |
|  | Average | 2.3 | 1 | 5.1 | 4.2 | 2.1 | 8.3 | 1.9 | -1.5 | 5.3 | 0.276 |
|  | Smaller than average | 11.5 | 5.1 | 25.7 | 4.8 | 0.7 | 33.2 | -6.7 | -19.9 | 6.5 | 0.319 |
|  | Very small | 2.5 | 0.3 | 17.7 | 12.1 | 2.7 | 53.4 | 9.7 | -9.1 | 28.5 | 0.313 |
|  | Do not know’ |  |  |  |  |  |  |  |  |  |  |
| Birth order | First born | 4.4 | 2.1 | 9.2 | 4.8 | 2.8 | 8.3 | 0.4 | -3.8 | 4.6 | 0.846 |
|  | 2–4 | 4.3 | 2.1 | 8.8 | 3.3 | 1.6 | 6.7 | -1 | -4.9 | 2.8 | 0.599 |
|  | Five or more | 4 | 0.9 | 16.9 | 2.1 | 0.3 | 14.9 | -1.9 | -9 | 5.1 | 0.592 |
| Mother’s parity | Primigravida | 6 | 3.2 | 11.1 | 4.5 | 2.7 | 7.2 | -1.5 | -5.8 | 2.8 | 0.495 |
|  | Multigravida | 2.5 | 1.1 | 5.6 | 3.1 | 1.3 | 7.3 | 0.6 | -2.8 | 3.9 | 0.738 |
| Preceding birth interval | >two years | 3.8 | 1.8 | 8 | 3.1 | 1.3 | 7.1 | -0.7 | -4.5 | 3.1 | 0.71 |
|  | First birth | 4.4 | 2.1 | 9.2 | 4.8 | 2.8 | 8.3 | 0.4 | -3.8 | 4.6 | 0.846 |
|  | ≤two years | 5.8 | 2.2 | 15.1 | 3.7 | 1.1 | 12.1 | -2.1 | -9.2 | 4.9 | 0.557 |
| Twin birth | No | 4.4 | 2.7 | 7.2 | 3.6 | 2.3 | 5.6 | -0.8 | -3.5 | 2 | 0.586 |
|  | Yes |  |  |  |  |  |  |  |  |  |  |
| Wanted last birth | Wanted then | 4.3 | 2.4 | 7.5 | 5.2 | 3.2 | 8.7 | 1 | -2.6 | 4.6 | 0.589 |
|  | Wanted later | 2.1 | 0.5 | 8.6 | 1.3 | 0.3 | 5.4 | -0.8 | -4.3 | 2.7 | 0.652 |
|  | Wanted no more | 8.5 | 3.1 | 23.2 | 12 | 3.4 | 41.3 | 3.6 | -13.7 | 20.8 | 0.686 |
| Time to health facility | <=30 minutes | 2.4 | 0.7 | 7.7 | 3.7 | 2.3 | 6.1 | 1.3 | -2 | 4.7 | 0.432 |
|  | >30 minutes | 0.6 | 0.1 | 3.9 | 4.7 | 2 | 10.8 | 4.1 | 0 | 8.2 | 0.049 |
| Birth attendants | Delivery without SBA | 3.1 | 1.4 | 7 | 8.9 | 3.4 | 23.5 | 5.8 | -3.2 | 14.8 | 0.208 |
|  | Delivery with SBA | 4.9 | 2.6 | 9.1 | 3.7 | 2 | 7 | -1.2 | -5 | 2.7 | 0.548 |
| Place of delivery | Home delivery | 3.6 | 1.6 | 8 | 9.5 | 3.6 | 24.6 | 5.8 | -3.7 | 15.3 | 0.23 |
|  | Public health facility | 3.9 | 1.8 | 8 | 4.4 | 2.2 | 8.7 | 0.5 | -3.6 | 4.6 | 0.807 |
|  | Private health facility | 6.4 | 1.9 | 21.9 | 1.4 | 0.4 | 5.9 | -5 | -13.2 | 3.2 | 0.23 |
| C-section past years | Caesarean | 3.7 | 0.9 | 15.7 | 2.6 | 0.6 | 10.8 | -1.1 | -7.6 | 5.4 | 0.734 |
|  | Not caesarean | 4.2 | 2.4 | 7.3 | 5.3 | 3 | 9.5 | 1.1 | -2.7 | 5 | 0.565 |
| ANC visits (three categories) | 1–3 visits | 2.5 | 0.6 | 10 | 4.3 | 0.6 | 30.3 | 1.8 | -7.3 | 11 | 0.694 |
|  | Four-plus visits | 1.3 | 0.5 | 3.7 | 2.5 | 1.2 | 5.5 | 1.2 | -1.2 | 3.6 | 0.314 |
|  | Do not know/None |  |  |  |  |  |  | 0 | 0 | 0 |  |
| ANC visits (two categories) | 0–3 visits | 2.6 | 0.8 | 8.4 | 3.8 | 0.5 | 26.4 | 1.1 | -6.9 | 9.1 | 0.785 |
|  | Four-plus visits | 1.3 | 0.5 | 3.7 | 2.5 | 1.2 | 5.5 | 1.2 | -1.2 | 3.6 | 0.315 |
| Days iron tablets taken | <180 days | 1.3 | 0.3 | 5.1 | 4.9 | 1.9 | 12.5 | 3.6 | -1.3 | 8.5 | 0.151 |
|  | 180-plus days | 1.4 | 0.4 | 4.5 | 1.9 | 0.6 | 6.3 | 0.5 | -2.3 | 3.3 | 0.722 |
| Newborn PNC within two days | No PNC | 1.1 | 0.3 | 4.3 | 4.6 | 1.4 | 14.5 | 3.5 | -2 | 9 | 0.208 |
|  | Yes PNC | 2.3 | 0.7 | 7.4 | 1.9 | 0.7 | 5.2 | -0.3 | -3.6 | 3 | 0.85 |
| Mother PNC within two days | No PNC | 1.1 | 0.3 | 4.3 | 3.2 | 0.8 | 13.5 | 2.2 | -2.7 | 7 | 0.381 |
|  | Yes PNC | 2.3 | 0.7 | 7.5 | 2.5 | 1.1 | 6 | 0.3 | -3.2 | 3.8 | 0.887 |
