# Supplementary material for: Elucidating trends and underlying drivers of neonatal mortality stagnation in Nepal: An analytical perspective on the 2016 and 2022 Demographic and Health Surveys
Source: PLoS One. 2025 Aug 22;20(8):e0330734. doi: 10.1371/journal.pone.0330734 (PMC12373174; doi:10.1371/journal.pone.0330734)
Supplement: S5 Table — (DOCX) [file pone.0330734.s005.docx]

S5 Table: The Neonatal Rates in the 2016 and 2022 NDHSs and the Difference Between Them, with Confidence Intervals for the Rates and the Difference and P-values for the Difference.

|  |  | **2016** | | | **2022** | | | **Change** | | |  |
| --- | --- | --- | --- | --- | --- | --- | --- | --- | --- | --- | --- |
| **Characteristics** | **Categories** | **NMR** | **95% LB** | **95% UB** | **NMR** | **95% LB** | **95% UB** | **NMR** | **NMR** | **95% LB** | **P-value for change** |
| National | National | 20.9 | 16.9 | 25.9 | 20.3 | 16.2 | 25.4 | -0.6 | -7 | 5.7 | 0.844 |
| Respondent’s language | Bhojpuri | 25.8 | 13 | 50.6 | 43 | 28.3 | 64.8 | 17.1 | -7.3 | 41.6 | 0.17 |
|  | Maithili | 23.4 | 15 | 36.2 | 27.4 | 16.3 | 45.8 | 4 | -13.3 | 21.4 | 0.651 |
|  | Nepali | 17.4 | 12.5 | 24 | 10.3 | 7.2 | 14.8 | -7 | -13.8 | -0.3 | 0.042 |
|  | Other | 22.3 | 14.7 | 33.4 | 24.7 | 16.5 | 37 | 2.5 | -11 | 16 | 0.718 |
| Ethnicity (three categories) | Advantaged | 18.4 | 13.4 | 25.2 | 19.7 | 14.6 | 26.5 | 1.3 | -6.9 | 9.5 | 0.755 |
|  | Disadvantaged Dalit | 27 | 17.2 | 42.1 | 26.3 | 17.1 | 40.1 | -0.7 | -17.2 | 15.7 | 0.931 |
|  | Disadvantaged Janajati | 20.9 | 13.8 | 31.6 | 15.8 | 9.8 | 25.2 | -5.1 | -16.5 | 6.3 | 0.376 |
| Ethnicity (two categories) | Advantaged | 18.3 | 12.4 | 26.8 | 10.7 | 6.9 | 16.6 | -7.6 | -16.1 | 0.8 | 0.077 |
|  | Disadvantaged | 22.1 | 17.2 | 28.4 | 23.9 | 18.7 | 30.6 | 1.8 | -6.3 | 9.9 | 0.658 |
| Wealth index in terciles | Poorer | 24.1 | 17.3 | 33.4 | 22.4 | 15.7 | 31.8 | -1.7 | -12.9 | 9.5 | 0.766 |
|  | Middle | 21.4 | 15.1 | 30.5 | 29.4 | 22.2 | 38.9 | 8 | -3.2 | 19.1 | 0.161 |
|  | Higher | 18 | 11.7 | 27.6 | 12 | 7.4 | 19.2 | -6 | -15.6 | 3.5 | 0.216 |
| Wealth index (one and two, three, four and five) | Middle | 15.8 | 8.7 | 28.4 | 22.7 | 13.8 | 36.9 | 6.9 | -7.6 | 21.4 | 0.353 |
|  | Poorer and poorest | 27.3 | 20.8 | 35.7 | 27.8 | 21.7 | 35.6 | 0.5 | -9.6 | 10.5 | 0.928 |
|  | Richer and richest | 16.4 | 10.7 | 25 | 8.7 | 4.8 | 15.6 | -7.7 | -16.3 | 0.9 | 0.079 |
| Province | Koshi | 22.8 | 12.6 | 41 | 26 | 15.4 | 43.8 | 3.2 | -15.8 | 22.2 | 0.739 |
|  | Madhesh | 24.3 | 16.1 | 36.6 | 29.7 | 20.6 | 42.6 | 5.4 | -9.2 | 19.9 | 0.469 |
|  | Bagmati | 10.9 | 5.1 | 23.1 | 11.2 | 4.8 | 26 | 0.3 | -12.2 | 12.7 | 0.967 |
|  | Gandaki |  |  |  |  |  |  | 0 | 0 | 0 |  |
|  | Lumbini | 18.4 | 11 | 30.7 | 17.2 | 9.8 | 30.1 | -1.2 | -14.6 | 12.2 | 0.861 |
|  | Karnali | 31.3 | 20.3 | 48.1 | 18.9 | 12 | 29.8 | -12.4 | -28.3 | 3.5 | 0.127 |
|  | Sudurpaschim | 29.9 | 17.7 | 50 | 17.8 | 9.7 | 32.3 | -12.1 | -30.7 | 6.6 | 0.204 |
| Ecological region | Hill | 16.4 | 10.8 | 24.7 | 13.2 | 9.1 | 19.2 | -3.1 | -11.5 | 5.3 | 0.467 |
|  | Mountain | 36.5 | 24.2 | 54.7 | 26.2 | 12.7 | 53.2 | -10.3 | -33.8 | 13.2 | 0.391 |
|  | Terai | 22 | 16.4 | 29.4 | 23.7 | 17.7 | 31.6 | 1.7 | -7.7 | 11 | 0.728 |
| Religion | Buddhist | 7.3 | 1 | 48.9 | 9.2 | 2.6 | 31.6 | 1.9 | -16 | 19.9 | 0.832 |
|  | Hindu | 22.5 | 18.1 | 28.1 | 19.4 | 15.3 | 24.5 | -3.1 | -9.9 | 3.6 | 0.36 |
|  | Muslim | 19.4 | 8 | 46.3 | 44 | 21 | 89.9 | 24.6 | -11.2 | 60.3 | 0.178 |
| Type of place | Rural | 26.3 | 19.7 | 35.1 | 24.1 | 18.5 | 31.4 | -2.2 | -12.1 | 7.7 | 0.667 |
|  | Urban | 16.3 | 11.9 | 22.4 | 18.2 | 13 | 25.4 | 1.9 | -6.2 | 9.9 | 0.65 |
| Size of household | <six members | 21.2 | 15.4 | 29.2 | 22.2 | 16.9 | 29.3 | 1 | -8.1 | 10.1 | 0.833 |
|  | ≥six members | 20.6 | 15.3 | 27.7 | 18 | 12.4 | 26 | -2.6 | -11.6 | 6.4 | 0.574 |
| Sex of household head | Female | 17.3 | 10.9 | 27.5 | 16.3 | 10.7 | 24.8 | -1 | -11.5 | 9.6 | 0.858 |
|  | Male | 22.4 | 17.6 | 28.4 | 22.1 | 17.1 | 28.4 | -0.3 | -8 | 7.4 | 0.942 |
| Indoor air pollution | No | 17.4 | 10.7 | 28.2 | 11.3 | 7.1 | 18.1 | -6.1 | -16 | 3.9 | 0.233 |
|  | Yes | 22.5 | 17.9 | 28.2 | 26.3 | 20.5 | 33.7 | 3.8 | -4.5 | 12.1 | 0.369 |
| Improved water and sanitation | Improved | 19.6 | 15 | 25.5 | 20.7 | 15.8 | 27 | 1.1 | -6.5 | 8.7 | 0.775 |
|  | Not a de jure resident | 38.0 | 21.5 | 66.4 | 13.5 | 5.5 | 32.3 | -24.5 | -49 | -0.1 | 0.049 |
|  | Unimproved | 19.3 | 12.6 | 29.4 | 21.9 | 12.9 | 37.1 | 2.6 | -11.5 | 16.8 | 0.714 |
| Maternal education | Basic (grades 1–8) | 23.3 | 14.4 | 37.8 | 25.3 | 18.4 | 34.7 | 2 | -11.9 | 15.8 | 0.78 |
|  | No education | 25.7 | 18.8 | 35.2 | 29.3 | 19.5 | 43.9 | 3.6 | -10.8 | 18 | 0.623 |
|  | Secondary and above (≥grade nine) | 16.1 | 10.9 | 23.7 | 11.2 | 7.5 | 16.7 | -4.9 | -12.6 | 2.8 | 0.208 |
| Maternal age (five categories) | 15–19 years | 33.2 | 17.1 | 63.6 | 29.3 | 15 | 56.3 | -3.9 | -33.1 | 25.2 | 0.792 |
|  | 20–24 years | 27.7 | 20.1 | 38 | 26.9 | 19.9 | 36.1 | -0.8 | -12.7 | 11.1 | 0.896 |
|  | 25–29 years | 16 | 10.8 | 23.6 | 20.2 | 13.8 | 29.4 | 4.2 | -5.6 | 14 | 0.405 |
|  | 30–34 years | 15 | 7.3 | 30.5 | 6.8 | 3 | 15.4 | -8.2 | -20.3 | 3.9 | 0.184 |
|  | 35 and above | 16.1 | 7.1 | 36.1 | 17.9 | 7.8 | 40.4 | 1.7 | -17.9 | 21.4 | 0.862 |
| Maternal age (three categories) | 15–19 years | 33.2 | 17.1 | 63.6 | 29.3 | 15 | 56.3 | -3.9 | -33.1 | 25.2 | 0.792 |
|  | 20–34 years | 20.3 | 15.9 | 25.9 | 20 | 15.6 | 25.5 | -0.3 | -7.3 | 6.6 | 0.922 |
|  | ≥35 years | 16.1 | 7.1 | 36.1 | 17.9 | 7.8 | 40.4 | 1.7 | -17.9 | 21.4 | 0.862 |
| Maternal use of tobacco | No | 21.3 | 17.1 | 26.5 | 20.5 | 16.2 | 25.8 | -0.8 | -7.5 | 5.8 | 0.805 |
|  | Yes | 14.5 | 7.3 | 28.6 | 16.8 | 8.2 | 33.8 | 2.3 | -13.1 | 17.6 | 0.773 |
| Maternal stature | <145 cm | 29.2 | 12.5 | 66.5 | 37.4 | 19.4 | 71 | 8.2 | -26.1 | 42.6 | 0.639 |
|  | ≥145 cm | 22.3 | 16.4 | 30.2 | 20.6 | 14.4 | 29.4 | -1.6 | -11.6 | 8.4 | 0.748 |
| Maternal anemia | Anemic | 29 | 19.7 | 42.3 | 21.8 | 14.3 | 33.1 | -7.1 | -21.5 | 7.2 | 0.328 |
|  | Not anemic | 17.8 | 10.9 | 29.2 | 23.7 | 15.9 | 35.2 | 5.8 | -7.1 | 18.7 | 0.375 |
| Owns mobile phone | No | 28.8 | 20.1 | 41.1 | 28 | 16.9 | 46.1 | -0.9 | -18.3 | 16.6 | 0.924 |
|  | Yes | 18.5 | 14.2 | 24.1 | 18.7 | 14.6 | 23.8 | 0.1 | -6.6 | 6.8 | 0.969 |
| Possesses a bank account | No | 22.9 | 17.7 | 29.6 | 27.5 | 21.4 | 35.3 | 4.6 | -4.5 | 13.6 | 0.323 |
|  | Yes | 17.2 | 11.8 | 25.1 | 9.6 | 6.3 | 14.4 | -7.6 | -15.2 | -0.1 | 0.048 |
| Internet use | Never used Internet | 22.5 | 18 | 28.2 | 31.3 | 23.5 | 41.6 | 8.8 | -1.5 | 19 | 0.093 |
|  | Used at some time | 13.7 | 7.8 | 23.9 | 14.6 | 10.4 | 20.3 | 0.9 | -8.2 | 10 | 0.847 |
| Empowerment: household decisions | No | 22.7 | 17.6 | 29.2 | 24.5 | 19 | 31.5 | 1.8 | -6.6 | 10.3 | 0.674 |
|  | Yes, can make decisions | 18 | 12.4 | 26.1 | 13 | 8.7 | 19.5 | -5 | -13.6 | 3.5 | 0.249 |
| Violence justified | Violence is not justified | 19.9 | 15.5 | 25.5 | 20.1 | 16 | 25.2 | 0.2 | -6.6 | 6.9 | 0.964 |
|  | Violence is justified | 23.3 | 15.9 | 34 | 21 | 11.9 | 37 | -2.2 | -17.1 | 12.6 | 0.767 |
| Empowerment: health care/family planning decisions | No | 23.4 | 18.8 | 29.2 | 25.9 | 19.1 | 35 | 2.5 | -6.9 | 11.9 | 0.605 |
|  | Yes | 10.2 | 5.1 | 20 | 16.4 | 12.3 | 21.8 | 6.2 | -2.1 | 14.6 | 0.143 |
| Newspaper/Magazine | At least once a week | 19.4 | 14.4 | 26.2 | 11.9 | 8.2 | 17.2 | -7.6 | -14.9 | -0.3 | 0.042 |
|  | Less than once a week | 22.6 | 16.7 | 30.4 | 27.1 | 20.9 | 35.2 | 4.6 | -5.2 | 14.3 | 0.359 |
| Radio/TV | Less than once a week | 22.5 | 16.6 | 30.3 | 26.6 | 20.5 | 34.5 | 4.1 | -5.5 | 13.8 | 0.403 |
|  | At least once a week | 19.5 | 14.4 | 26.4 | 12.2 | 8.4 | 17.6 | -7.3 | -14.8 | 0.1 | 0.052 |
| Knows about ’HMG | No | 21.7 | 16.7 | 28.1 | 20.2 | 15 | 27.2 | -1.4 | -9.7 | 6.8 | 0.732 |
|  | Yes | 19.4 | 13.7 | 27.4 | 20.4 | 14.7 | 28.2 | 1 | -8.5 | 10.4 | 0.837 |
| Husband’s education | Basic (grades 1–8) | 29.9 | 20.3 | 43.8 | 21.5 | 15.5 | 29.7 | -8.4 | -21.9 | 5 | 0.218 |
|  | No education/Do not know | 19.5 | 11.3 | 33.4 | 37.5 | 23.5 | 59.3 | 18 | -2.3 | 38.3 | 0.082 |
|  | Secondary and above (≥grade nine) | 18.2 | 13.4 | 24.6 | 14.1 | 9.6 | 20.7 | -4.1 | -11.8 | 3.7 | 0.303 |
| Husband’s occupation (four categories) | Agriculture | 30.6 | 21.2 | 44 | 12.5 | 7.2 | 21.5 | -18.1 | -31.2 | -5.1 | 0.006 |
|  | Manual (skilled/unskilled) | 20.7 | 14.5 | 29.3 | 25.9 | 19.7 | 34 | 5.3 | -4.8 | 15.4 | 0.306 |
|  | Sales, clerical, other | 18.5 | 13.1 | 26.1 | 15.4 | 10.4 | 22.9 | -3.1 | -11.9 | 5.8 | 0.497 |
| Birthweight taken | Not taken | 33.2 | 25.3 | 43.5 | 44.9 | 27.1 | 73.5 | 11.7 | -12.4 | 35.8 | 0.342 |
|  | Yes, taken | 12.5 | 8.9 | 17.7 | 12.3 | 8.1 | 18.5 | -0.3 | -6.9 | 6.3 | 0.934 |
| Sex of child | Female | 17.3 | 12.2 | 24.6 | 16.3 | 11.3 | 23.4 | -1 | -9.5 | 7.4 | 0.814 |
|  | Male | 24.2 | 18.4 | 31.7 | 23.8 | 18 | 31.4 | -0.3 | -9.7 | 9 | 0.943 |
| Birthweight | Large (≥3,500 g) | 11 | 5.8 | 20.8 | 6.9 | 2.5 | 19.2 | -4.1 | -14.1 | 5.9 | 0.42 |
|  | Normal (2,500–3,500 g) | 9.8 | 5.9 | 16.1 | 12.4 | 7.2 | 21.1 | 2.6 | -5.6 | 10.8 | 0.538 |
|  | Not weighed or do not know | 33.2 | 25.3 | 43.5 | 44.9 | 27.1 | 73.5 | 11.7 | -12.4 | 35.8 | 0.342 |
|  | Small (<2,500 g) | 27.2 | 14.1 | 51.9 | 24.2 | 11.7 | 49.5 | -3 | -27.9 | 21.9 | 0.813 |
| Perceived birthweight | Very large | 26.5 | 10.5 | 65.2 | 21.2 | 4.9 | 86.9 | -5.3 | -44.1 | 33.5 | 0.789 |
|  | Larger than average | 32.2 | 20.1 | 51.3 | 11.2 | 4.2 | 29.4 | -21.0 | -39.6 | -2.4 | 0.027 |
|  | Average | 13 | 9.5 | 18 | 19.1 | 13.2 | 27.6 | 6.1 | -2.1 | 14.3 | 0.147 |
|  | Smaller than average | 34.9 | 21.8 | 55.5 | 12.5 | 4.5 | 34.3 | -22.4 | -43.1 | -1.8 | 0.033 |
|  | Very small | 35.2 | 17.8 | 68.5 | 37.5 | 16.3 | 83.9 | 2.3 | -36.5 | 41 | 0.908 |
| Birth order | First born | 24.3 | 18.1 | 32.7 | 24.6 | 18.4 | 32.7 | 0.2 | -9.9 | 10.3 | 0.967 |
|  | 2–4 | 17.6 | 12.9 | 23.9 | 16.3 | 11.5 | 23.1 | -1.2 | -9.1 | 6.6 | 0.755 |
|  | Five or more | 25.5 | 14.2 | 45.2 | 30.2 | 14.4 | 62.5 | 4.8 | -21.8 | 31.4 | 0.726 |
| Mother’s parity | Primigravida | 25.6 | 19.3 | 33.8 | 24 | 18.4 | 31.4 | -1.5 | -11.1 | 8.1 | 0.757 |
|  | Multigravida | 15.7 | 11.4 | 21.6 | 15.3 | 10.4 | 22.5 | -0.3 | -8.1 | 7.4 | 0.93 |
| Preceding birth interval | >two years | 14.6 | 10.3 | 20.8 | 11.5 | 7.6 | 17.2 | -3.2 | -10.1 | 3.8 | 0.371 |
|  | First birth | 24.3 | 18.1 | 32.7 | 24.6 | 18.4 | 32.7 | 0.2 | -9.9 | 10.3 | 0.967 |
|  | ≤two years | 29.4 | 18 | 47.9 | 37.7 | 22.6 | 62.4 | 8.3 | -15.7 | 32.3 | 0.498 |
| Twin birth | No | 20.1 | 16.1 | 25 | 20.1 | 16 | 25.2 | 0 | -6.3 | 6.4 | 0.988 |
|  | Yes | 74.3 | 21.4 | 227.5 | 10.7 | 1.4 | 76.2 | -63.6 | -152.8 | 25.6 | 0.162 |
| Wanted last birth | Wanted then | 21.2 | 16.7 | 27 | 26.5 | 20.3 | 34.5 | 5.2 | -3.4 | 13.9 | 0.236 |
|  | Wanted later | 17.7 | 9.6 | 32.3 | 13.2 | 6.4 | 27.2 | -4.5 | -18.9 | 9.8 | 0.538 |
|  | Wanted no more | 24.9 | 13.8 | 44.7 | 28.6 | 13.2 | 60.5 | 3.6 | -22.5 | 29.8 | 0.785 |
| Time to health facility | <=30 minutes | 4.2 | 1.7 | 10.3 | 19.1 | 15 | 24.4 | 15 | 9 | 21 | 0 |
|  | >30 minutes | 9.8 | 5.4 | 17.6 | 27.5 | 16.4 | 45.6 | 17.7 | 2.6 | 32.8 | 0.022 |
| Birth attendants | Delivery without SBA | 25.7 | 19.1 | 34.5 | 36.1 | 20.3 | 63.4 | 10.4 | -11.5 | 32.3 | 0.352 |
|  | Delivery with SBA | 16.9 | 12.4 | 22.8 | 14.3 | 9.9 | 20.7 | -2.5 | -9.9 | 4.8 | 0.498 |
| Place of delivery | Home delivery | 25.9 | 19.1 | 35.2 | 35.9 | 19.7 | 64.6 | 10 | -12.8 | 32.7 | 0.391 |
|  | Public health facility | 14.9 | 10.4 | 21.4 | 14.1 | 9 | 22 | -0.8 | -9.1 | 7.5 | 0.848 |
|  | Private health facility | 21.9 | 12.8 | 37.3 | 16.1 | 8.7 | 29.7 | -5.8 | -21.2 | 9.5 | 0.455 |
| C-section past pregnancy | Caesarean | 8.3 | 3 | 22.6 | 9.3 | 3.6 | 23.4 | 1 | -11 | 13 | 0.874 |
|  | Not caesarean | 21.8 | 17.4 | 27.3 | 21.1 | 15.1 | 29.4 | -0.7 | -9.3 | 7.8 | 0.866 |
| ANC visits (three categories) | 1–3 visits | 20.9 | 12.8 | 34 | 25.7 | 13.1 | 49.8 | 4.8 | -15.2 | 24.7 | 0.64 |
|  | Four-plus visits | 6.1 | 3.7 | 10.1 | 11.1 | 7.1 | 17.2 | 5 | -0.8 | 10.7 | 0.089 |
|  | Do not know/None | 17.3 | 6.5 | 45.1 | 7.1 | 1 | 49.2 | -10.2 | -31.8 | 11.5 | 0.358 |
| ANC visits (two categories) | 0–3 visits | 20.2 | 13.2 | 30.9 | 23.3 | 12.1 | 44.3 | 3 | -14.4 | 20.4 | 0.732 |
|  | Four-plus visits | 6.1 | 3.7 | 10.1 | 11.1 | 7.1 | 17.2 | 5 | -0.8 | 10.7 | 0.09 |
| Days iron tablets taken | <180 days | 14.4 | 9.4 | 22.2 | 19.4 | 11.6 | 32.5 | 5 | -6.8 | 16.8 | 0.406 |
|  | 180-plus days | 5.7 | 2.9 | 11 | 10.9 | 6.5 | 18.1 | 5.2 | -1.6 | 11.9 | 0.132 |
| Newborn PNC within two days | No PNC | 14.6 | 9.6 | 22.2 | 26.3 | 16 | 42.9 | 11.6 | -2.7 | 26 | 0.112 |
|  | Yes PNC | 7 | 4 | 12.2 | 7.7 | 4.4 | 13.4 | 0.7 | -5.1 | 6.5 | 0.807 |
| Mother PNC within two days | No PNC | 11.4 | 7.2 | 17.8 | 19.4 | 11 | 34.1 | 8 | -4.1 | 20.2 | 0.194 |
|  | Yes PNC | 9.7 | 5.8 | 16.1 | 10.6 | 6.5 | 17.3 | 0.9 | -6.2 | 8.1 | 0.796 |
