# Supplementary material for: Elucidating trends and underlying drivers of neonatal mortality stagnation in Nepal: An analytical perspective on the 2016 and 2022 Demographic and Health Surveys
Source: PLoS One. 2025 Aug 22;20(8):e0330734. doi: 10.1371/journal.pone.0330734 (PMC12373174; doi:10.1371/journal.pone.0330734)
Supplement: S6 Table — (DOCX) [file pone.0330734.s006.docx]

S6 Table : The P-values for the Differences Between the 2016 and 2022 NDHSs in the Early Neonatal, Late Neonatal, and NMRs for all Categories of the Covariates, with Symbols for the Level of Significance.

| **Characteristics** | **Categories** | **P-value for ENMR difference** | | **P-value for LNMR difference** | | **P-value for NMR difference** | |
| --- | --- | --- | --- | --- | --- | --- | --- |
| National | National | 0.954 | NS | 0.737 | NS | 0.844 | NS |
| Respondent’s language | Bhojpuri | 0.03 | + | 0.25 | NS | 0.17 | NS |
|  | Maithili | 0.401 | NS | 0.61 | NS | 0.651 | NS |
|  | Nepali | 0.005 | -- | 0.379 | NS | 0.042 | - |
|  | Other | 0.727 | NS | 0.953 | NS | 0.718 | NS |
| Ethnicity (three categories) | Advantaged | 0.951 | NS | 0.609 | NS | 0.755 | NS |
|  | Disadvantaged Dalit | 0.77 | NS | 0.302 | NS | 0.931 | NS |
|  | Disadvantaged Janajati | 0.456 | NS | 0.654 | NS | 0.376 | NS |
| Ethnicity (two categories) | Advantaged | 0.043 | - | 0.843 | NS | 0.077 | NS |
|  | Disadvantaged | 0.526 | NS | 0.749 | NS | 0.658 | NS |
| Wealth index in terciles | Poorer | 0.616 | NS | 0.752 | NS | 0.766 | NS |
|  | Middle | 0.33 | NS | 0.26 | NS | 0.161 | NS |
|  | Higher | 0.603 | NS | 0.081 | NS | 0.216 | NS |
| Wealth index (one and two, three, four and five) | Middle | 0.297 | NS | 0.82 | NS | 0.353 | NS |
|  | Poorer and poorest | 0.81 | NS | 0.525 | NS | 0.928 | NS |
|  | Richer and richest | 0.254 | NS | 0.104 | NS | 0.079 | NS |
| Province | Koshi | 0.486 | NS | 0.412 | NS | 0.739 | NS |
|  | Madhesh | 0.215 | NS | 0.532 | NS | 0.469 | NS |
|  | Bagmati | 0.967 | NS |  |  | 0.967 | NS |
|  | Gandaki |  |  |  |  |  | NS |
|  | Lumbini | 0.691 | NS | 0.718 | NS | 0.861 | NS |
|  | Karnali | 0.053 | NS | 0.693 | NS | 0.127 | NS |
|  | Sudurpaschim | 0.057 | NS | 0.239 | NS | 0.204 | NS |
| Ecological region | Hill | 0.395 | NS | 0.776 | NS | 0.467 | NS |
|  | Mountain | 0.467 | NS | 0.751 | NS | 0.391 | NS |
|  | Terai | 0.525 | NS | 0.655 | NS | 0.728 | NS |
| Religion | Buddhist | 0.995 | NS | 0 | --- | 0.832 | NS |
|  | Hindu | 0.425 | NS | 0.664 | NS | 0.36 | NS |
|  | Muslim | 0.155 | NS | 0.893 | NS | 0.178 | NS |
| Type of place | Rural | 0.633 | NS | 0.978 | NS | 0.667 | NS |
|  | Urban | 0.518 | NS | 0.751 | NS | 0.65 | NS |
| Size of household | <six members | 0.853 | NS | 0.92 | NS | 0.833 | NS |
|  | ≥six members | 0.681 | NS | 0.646 | NS | 0.574 | NS |
| Sex of household head | Female | 0.669 | NS | 0.47 | NS | 0.858 | NS |
|  | Male | 0.819 | NS | 0.544 | NS | 0.942 | NS |
| Indoor air pollution | No | 0.639 | NS | 0.123 | NS | 0.233 | NS |
|  | Yes | 0.58 | NS | 0.332 | NS | 0.369 | NS |
| Improved water and sanitation | Improved | 0.956 | NS | 0.379 | NS | 0.775 | NS |
|  | Not a de jure resident | 0.177 | NS | 0.184 | NS | 0.049 | - |
|  | Unimproved | 0.375 | NS | 0.221 | NS | 0.714 | NS |
| Maternal education | Basic (grades 1–8) | 0.708 | NS | 0.039 | + | 0.78 | NS |
|  | No education | 0.389 | NS | 0.428 | NS | 0.623 | NS |
|  | Secondary and above (≥grade nine) | 0.38 | NS | 0.268 | NS | 0.208 | NS |
| Maternal age (five categories) | 15–19 years | 0.625 | NS | 0.621 | NS | 0.792 | NS |
|  | 20–24 years | 0.667 | NS | 0.289 | NS | 0.896 | NS |
|  | 25–29 years | 0.454 | NS | 0.747 | NS | 0.405 | NS |
|  | 30–34 years | 0.045 | - | 0.453 | NS | 0.184 | NS |
|  | 35 and above | 0.724 | NS | 0.556 | NS | 0.862 | NS |
| Maternal age (three categories) | 15–19 years | 0.625 | NS | 0.621 | NS | 0.792 | NS |
|  | 20–34 years | 0.951 | NS | 0.727 | NS | 0.922 | NS |
|  | ≥35 years | 0.724 | NS | 0.556 | NS | 0.862 | NS |
| Maternal use of tobacco | No | 0.91 | NS | 0.738 | NS | 0.805 | NS |
|  | Yes | 0.728 | NS | 0.894 | NS | 0.773 | NS |
| Maternal stature | <145 cm | 0.161 | NS | 0.189 | NS | 0.639 | NS |
|  | ≥145 cm | 0.654 | NS | 0.784 | NS | 0.748 | NS |
| Maternal anemia | Anemic | 0.454 | NS | 0.393 | NS | 0.328 | NS |
|  | Not anemic | 0.303 | NS | 0.987 | NS | 0.375 | NS |
| Owns mobile phone | No | 0.592 | NS | 0.176 | NS | 0.924 | NS |
|  | Yes | 0.789 | NS | 0.489 | NS | 0.969 | NS |
| Possesses a bank account | No | 0.184 | NS | 0.573 | NS | 0.323 | NS |
|  | Yes | 0.017 | - | 0.568 | NS | 0.048 | - |
| Internet use | Never used Internet | 0.104 | NS | 0.616 | NS | 0.093 | NS |
|  | Used at some time | 0.895 | NS | 0.867 | NS | 0.847 | NS |
| Empowerment: household decisions | No | 0.553 | NS | 0.792 | NS | 0.674 | NS |
|  | Yes, can make decisions | 0.247 | NS | 0.814 | NS | 0.249 | NS |
| Violence justified | Violence is not justified | 0.961 | NS | 0.838 | NS | 0.964 | NS |
|  | Violence is justified | 0.931 | NS | 0.331 | NS | 0.767 | NS |
| Empowerment: health care/family planning decisions | No | 0.967 | NS | 0.279 | NS | 0.605 | NS |
|  | Yes | 0.005 | ++ | 0.266 | NS | 0.143 | NS |
| Newspaper/Magazine | At least once a week | 0.023 | - | 0.806 | NS | 0.042 | - |
|  | Less than once a week | 0.269 | NS | 0.828 | NS | 0.359 | NS |
| Radio/TV | Less than once a week | 0.303 | NS | 0.804 | NS | 0.403 | NS |
|  | At least once a week | 0.028 | - | 0.843 | NS | 0.052 | NS |
| Knows about HMG | No | 0.993 | NS | 0.417 | NS | 0.732 | NS |
|  | Yes | 0.885 | NS | 0.514 | NS | 0.837 | NS |
| Husband’s education | Basic (grades 1–8) | 0.157 | NS | 0.789 | NS | 0.218 | NS |
|  | No education/Do not know | 0.087 | NS | 0.622 | NS | 0.082 | NS |
|  | Secondary and above (≥grade nine) | 0.548 | NS | 0.263 | NS | 0.303 | NS |
| Husband’s occupation (four categories) | Agriculture | 0.021 | - | 0.12 | NS | 0.006 | -- |
|  | Manual (skilled/unskilled) | 0.345 | NS | 0.753 | NS | 0.306 | NS |
|  | Not working | 0 | --- |  |  | 0 | --- |
|  | Sales, clerical, other | 0.48 | NS | 0.909 | NS | 0.497 | NS |
| Birthweight taken | Not taken | 0.53 | NS | 0.312 | NS | 0.342 | NS |
|  | Yes, taken | 0.893 | NS | 0.742 | NS | 0.934 | NS |
| Sex of child | Female | 0.673 | NS | 0.754 | NS | 0.814 | NS |
|  | Male | 0.809 | NS | 0.516 | NS | 0.943 | NS |
| Birthweight | Large (≥3,500 g) | 0.435 | NS | 0.779 | NS | 0.42 | NS |
|  | Normal (2,500–3,500 g) | 0.114 | NS | 0.18 | NS | 0.538 | NS |
|  | Not weighed or do not know | 0.53 | NS | 0.312 | NS | 0.342 | NS |
|  | Small (<2,500 g) | 0.1 | NS | 0.186 | NS | 0.813 | NS |
| Perceived birthweight | Very large | 0.789 | NS |  |  | 0.789 | NS |
|  | Larger than average | 0.007 | -- | 0.84 | NS | 0.027 | - |
|  | Average | 0.268 | NS | 0.276 | NS | 0.147 | NS |
|  | Smaller than average | 0.062 | NS | 0.319 | NS | 0.033 | - |
|  | Very small | 0.669 | NS | 0.313 | NS | 0.908 | NS |
|  | Do not know | 0.55 | NS |  |  | 0.55 | NS |
| Birth order | First born | 0.967 | NS | 0.846 | NS | 0.967 | NS |
|  | 2–4 | 0.953 | NS | 0.599 | NS | 0.755 | NS |
|  | Five or more | 0.612 | NS | 0.592 | NS | 0.726 | NS |
| Mother’s parity | Primigravida | 0.995 | NS | 0.495 | NS | 0.757 | NS |
|  | Multigravida | 0.804 | NS | 0.738 | NS | 0.93 | NS |
| Preceding birth interval | >two years | 0.426 | NS | 0.71 | NS | 0.371 | NS |
|  | First birth | 0.967 | NS | 0.846 | NS | 0.967 | NS |
|  | ≤two years | 0.371 | NS | 0.557 | NS | 0.498 | NS |
| Twin birth | No | 0.784 | NS | 0.586 | NS | 0.988 | NS |
|  | Yes | 0.162 | NS |  |  | 0.162 | NS |
| Wanted last birth | Wanted then | 0.303 | NS | 0.589 | NS | 0.236 | NS |
|  | Wanted later | 0.604 | NS | 0.652 | NS | 0.538 | NS |
|  | Wanted no more | 0.994 | NS | 0.686 | NS | 0.785 | NS |
| Time to health facility | <=30 minutes | 0 | +++ | 0.432 | NS | 0 | +++ |
|  | >30 minutes | 0.067 | NS | 0.049 | + | 0.022 | + |
| Birth attendants | Delivery without SBA | 0.659 | NS | 0.208 | NS | 0.352 | NS |
|  | Delivery with SBA | 0.671 | NS | 0.548 | NS | 0.498 | NS |
| Place of delivery | Home delivery | 0.699 | NS | 0.23 | NS | 0.391 | NS |
|  | Public health facility | 0.719 | NS | 0.807 | NS | 0.848 | NS |
|  | Private health facility | 0.899 | NS | 0.23 | NS | 0.455 | NS |
| C-section past years | Caesarean | 0.683 | NS | 0.734 | NS | 0.874 | NS |
|  | Not caesarean | 0.634 | NS | 0.565 | NS | 0.866 | NS |
| ANC visits (three categories) | 1–3 visits | 0.75 | NS | 0.694 | NS | 0.64 | NS |
|  | Four-plus visits | 0.163 | NS | 0.314 | NS | 0.089 | NS |
|  | Do not know/None | 0.516 | NS |  |  | 0.358 | NS |
| ANC visits (two categories) | 0–3 visits | 0.81 | NS | 0.785 | NS | 0.732 | NS |
|  | Four-plus visits | 0.163 | NS | 0.315 | NS | 0.09 | NS |
| Days iron tablets taken | <180 days | 0.802 | NS | 0.151 | NS | 0.406 | NS |
|  | 180-plus days | 0.14 | NS | 0.722 | NS | 0.132 | NS |
| Newborn PNC within two days | No PNC | 0.238 | NS | 0.208 | NS | 0.112 | NS |
|  | Yes PNC | 0.675 | NS | 0.85 | NS | 0.807 | NS |
| Mother PNC within two days | No PNC | 0.306 | NS | 0.381 | NS | 0.194 | NS |
|  | Yes PNC | 0.83 | NS | 0.887 | NS | 0.796 | NS |
