# Supplementary material for: Elucidating trends and underlying drivers of neonatal mortality stagnation in Nepal: An analytical perspective on the 2016 and 2022 Demographic and Health Surveys
Source: PLoS One. 2025 Aug 22;20(8):e0330734. doi: 10.1371/journal.pone.0330734 (PMC12373174; doi:10.1371/journal.pone.0330734)
Supplement: S7 Table — (DOCX) [file pone.0330734.s007.docx]

S7 Table: Study Variables Included in the Analysis for the 2022 NDHS.

| **Variables** | **Definition** | **Category (reference coding)** |
| --- | --- | --- |
| **Outcome variables** | NMR: Death of neonates in the first month of life (0–30 days) per 1,000 live births within the period 1–59 months preceding the survey | - High (>25 per 1,000) - Low (<15 per 1,000) |
| **Independent variables** | | |
| **Household characteristics** | | |
| Language | The respondent’s native language | - Nepali - Maithili - Bhojpuri - Others (Newari, Gurung, Magar) |
| Ethnicity | Ethnic categorization (see above) | - Advantaged - Disadvantaged Dalit - Disadvantaged Janajati |
| Wealth index | Quintile based on household wealth index score | - Poorer and poorest - Middle - Richer and richest |
| Province | Province | - Koshi - Madhesh - Bagmati - Gandaki - Lumbini - Karnali - Sudurpaschim |
| Residence | Rural and urban | - Rural - Urban |
| Eco region | Eco region | - Terai - Hill - Mountain |
| Religion | The respondent’s religion | - Hindu - Buddhist - Muslim - Kirat - Cristian - Other |
| Indoor air pollution | Cooking inside the home using solid fuel | - Yes - No |
| Improved water and sanitation | Sanitation: households with access to both improved drinking water and improved toilet facility | - Yes - No |
| **Maternal characteristics** | | |
| Mother’s education | Level of education is classified as the years of schooling/grades completed | - No education - Basic education (grades 1–8) - Secondary and above (≥grade nine) |
| Maternal age | Age of the mother in years | - 15–19 - 20–24 - 25–29 - 29–33 - 20–34 - 35 and above |
| Maternal employment | Cash earning from the job | - Not working - Agriculture - Manual labor (skilled/unskilled) - Paid Job |
| Body mass index | Body mass index of the mother (Kg/m^2^) | - Normal (18.5–25) - Less (<18.5) - Overweight/Obese (≥25) |
| Anaemia | <12 g/dl for non-pregnant and <11 g/dl for pregnant women | - Yes - No |
| Stature | Stature of the mother | - Normal (>145 cm) - Less (145 cm) |
| Tobacco use | Tobacco use habit of the respondent | - Yes - No |
| Alcohol use | Alcohol use at some point in time | - Yes - No |
| **Husband’s characteristics** | | |
| Husband’s alcohol use | Alcohol use at some point in time | - Yes - No |
| Husband’s employment | Cash earning from the job | - Not working - Agriculture - Manual labor (skilled/unskilled) - Working paid |
| Husband’s education | Level of education is classified as the years of schooling/grades completed | - No education - Basic education (grades 1–8) - Secondary and above (≥grade nine) |
| **Access related variables** | | |
| Access to a bank account | Possesses a bank account | - Yes - No |
| Has a mobile phone | Owns mobile phone | - Yes - No |
| Internet use | Use of Internet at some point in time | - Yes - No |
| Exposure to health programs in media | Heard or seen at least two health programs in media | - Yes - No |
| Attended HMG meeting | Attended in the HMG meeting at least once | - Yes - No |
| Distance to health facility | Duration to reach the nearest health facility in minutes | - >30 mins - <30 mins |
| Problems in accessing health care services | Problems in accessing health care services is classified as whether the women perceived the following problems: getting permission to go for treatment (medical help), getting money needed for treatment, distance to health facility, not wanting to go alone to the health facility, concern of no female health provider | - All four problems - Some (1–3) - None |
| **Women’s empowerments and domestic violence** | | |
| Empowerment in household decision-making | Women’s position in decision-making in the household | - Can make decisions - Cannot make decisions |
| Domestic violence | Experienced any spousal violence (beating, burning, or arguing) | - Yes - No |
| Decision-making on health service use | Mother’s decision-making ability for health service use | - Yes - No |
| Awareness on HMG | Awareness of HMGs in the respective wards | - No - Yes |
| **Health system-related factors** | | |
| Number of ANC visits | ANC visits times | - >=four times - <=four times - Do not know |
| Preceding birth interval | Birth interval of the women in recent birth | - >two years - ≤two years - First birth |
| Iron tables/syrup during pregnancy | Days of iron tablets taken | - >=180 days - <180 days/Not taken/Do not know |
| Perceived problem not having female providers | Classified as if having no female health care provider is a perceived problem | - No problem   Big problem |
| **Birth characteristics** | | |
| Sex of the child | Sex of the child | - Male - female |
| Place of delivery | Place and type of delivery | - Home delivery - Public health facility   Private health facility |
| Mode of delivery | Delivery procedure | - Non-caesarean   Caesarean |
| Attendant at delivery | Delivery attended by SBA | - Yes   No |
| Twin birth | Whether the pregnancy ended as twin birth | - Yes   No |
| Birthweight | Weight of the newborn | - Small (<25,00 gm) - Normal (2,500–3500 gm) - Large (≥3,500 gm) - Not weighted at birth/Do not know |
| Perceived birthweight | Birthweight perceived by the mother | - Very small - Smaller than average - Very large - Do not know |
| Postnatal check-up for mother | PNC provided to mothers | - Yes - No |
| Postnatal check-up for neonate | Postnatal check-up done for neonate | - Yes - No |
| **Pregnancy-related characteristics** | | |
| Wanted pregnancy | Woman wanted the recent pregnancy | - Wanted then - Wanted later - Wanted no more |
| Birth spacing | Birth spacing of the child | - None - 1–2 years - More than two years |
| Birth order | Birth order of the last child born | - First - Second - Third or higher |
