# Supplementary material for: Elucidating trends and underlying drivers of neonatal mortality stagnation in Nepal: An analytical perspective on the 2016 and 2022 Demographic and Health Surveys
Source: PLoS One. 2025 Aug 22;20(8):e0330734. doi: 10.1371/journal.pone.0330734 (PMC12373174; doi:10.1371/journal.pone.0330734)
Supplement: Attachment — Submitted filename: Reviewer 2 comments.docx [file pone.0330734.s008.docx]

Overall Comments:
This is a very important paper highlighting the trends, status, and determinants of neonatal deaths in Nepal.

Detailed Comments:

1. Abbreviations and Punctuation:
   - Revisit the use of abbreviations throughout the document to ensure consistency and clarity.
   - Review punctuation for correctness and coherence across the text.
2. Abstract and Conclusion:
   - In the last line, suggest adding that in addition to exploring determinants, rigorous studies on the causes of neonatal deaths are necessary.
3. Page 1:
   - In the relevant paragraph, suggest including “2030” to indicate the Sustainable Development Goal (SDG) target year.
4. Page 2:
   - Expand MNH (Maternal and Newborn Health) at its first use.
5. Page 3:
   - Expand the following abbreviations at first use:
     - SDIP
     - CB-NCP
     - IMNCI
6. Page 4 – Methods Section:
   - After the sentence: *“The NDHSs is a nationally representative household.”*, suggest adding a reference:
     *“Detailed information for the household and health facility survey sample designs, sample selection, and sample weighting are available in the final reports.”*
7. Page 5 – Study Variables:
   - The sentence *“The NMR is an indicator published in the NDHS final reports and on STATcompiler. [22]”* is unclear. Suggest rephrasing for clarity.
   - Clarify whether neonatal mortality refers to deaths within 30 days or 28 days. The sentence suggests both:
     *“Both of these sources use the standard DHS calculation for NMR based on neonatal deaths in the first month of life, or 30 days.”* — verify accuracy.
8. Page 7 – Ethical Considerations:
   - Clarify whether a separate ethical approval was obtained. If not, suggest writing that t*he NDHSs obtained ethical approvals from the NHR and a separate approval was not sought for this study based on secondary analysis of the NDHSs.*
9. Page 8:
   - Expand ND (Neonatal Deaths) at first use.
10. Page 14:

- The sentence is unclear:
  *“While the association between ANC care and background variables is explored in Chapter 9 of the final report for both the 2016 and 2022 surveys, detailed articulation of these pathways is constrained by low statistical power in this analysis.”*
  - Which report and which chapter are being referenced? Please clarify.

11. Page 16:

- Consider simplifying the description of federalism. For example, clarify where municipalities belong within the three tiers (federal, provincial, local).

12. Page 17:

- Recommend comparing NMR with the SDG 2030 target, rather than the NeNAP target, for consistency and relevance to international readers.

13. Page 19:

- The phrase *“maternal and husband”* should likely be revised to *“maternal and paternal.”*

14. Page 20:

- Expand HeMG (Health Mothers’ Group) at first use.

15. Conclusions and Key Recommendations:

- This section reads like Conclusions only. Recommendations can go into the Discussion section. Suggest strengthening this section by linking conclusions more explicitly to SDG targets.

16. Study Limitations:

- Recommend including a brief discussion of the limitations of the study.

17. Results and Discussion:

- Consider shortening these sections to maintain the reader’s engagement.

18. Table 2:

- Suggest replacing the term “respondent” with more appropriate terminology.
- Clarify if “husband” refers to “father” in the context of the study.

19. Figures:

- Figures 1 and 2 are blurred and difficult to read — suggest improving image resolution.
- Figure 3: Confirm if the “Late Neonatal Period” is 0–28 days or 0–27 days. Please verify.
- Ensure consistent font size and type across all figures and graphs.
