# Supplementary material for: Elucidating trends and underlying drivers of neonatal mortality stagnation in Nepal: An analytical perspective on the 2016 and 2022 Demographic and Health Surveys
Source: PLoS One. 2025 Aug 22;20(8):e0330734. doi: 10.1371/journal.pone.0330734 (PMC12373174; doi:10.1371/journal.pone.0330734)
Supplement: Attachment — Submitted filename: Comments and response final.docx [file pone.0330734.s010.docx]

Deare Editor,

Kindly find the Reponses of the revision based on the comments provided by the reviewers.

| **Reviewer’s comment** | **Response** | **Page#, line#** |
| --- | --- | --- |
| Reviewer 1: This work studied trends and the key determinants of the NMR (Neonatal Mortality Rate) in Nepal, using NDHS 2016 and NDHS 2022 dat. data (Nepal Demographic and Health Surveys). It has great importance for the medical and public health fields and can contribute to medical and governmental decision-making. About 43 independent variables were analysed and organized in 8 groups.  The authors collected and analyzed all data using appropriated methodology. They also applied appropriate methodology for analysis and discussion and reflection on the data. They also addressed that the data show the need for adaptation of medical and hospital practices, as well as public health. | Thank you. We greatly appreciate your comments. It is encouraging note to any authors. |  |
| Abbreviations and Punctuation: Revisit the use of abbreviations throughout the document to ensure consistency and clarity. Review punctuation for correctness and coherence across the text. |  |  |
| **Abstract and conclusion** |  |  |
| o In the last line, suggest adding that in addition to exploring determinants, rigorous studies on the causes of neonatal deaths are necessary. | While determinants have been explored, further targeted studies are warranted to uncover the causes of neonatal deaths in Nepal. | See page #3, lines #8-9 |
| 3. Page 1: o In the relevant paragraph, suggest including “2030” to indicate the Sustainable Development Goal (SDG) target year. | Added as - by 2030. | See page #4 line #18 |
| 4. Page 2: o Expand MNH (Maternal and Newborn Health) at its first use. | Added as advised. | See page #5 line #12 |
| 5. Page 3: o Expand the following abbreviations at first use: ♣ SDIP ♣ CB-NCP ♣ IMNCI | Added SDIP=Safe Delivery Incentive Programme  CB-NCP= Community-based Newborn Care Program  IMNCI=Integrated Management of Neonatal and Childhood Illness | Page #6, lines #12,#13, #14 |
| 6. Page 4 – Methods Section: o After the sentence: “The NDHSs is a nationally representative household.”, suggest adding a reference: “Detailed information for the household and health facility survey sample designs, sample selection, and sample weighting are available in the final reports.” | Added the reference as suggested | Page #7, line #21 |
| Page 5 – Study Variables: o The sentence “The NMR is an indicator published in the NDHS final reports and on STATcompiler. [22]” is unclear. Suggest rephrasing for clarity.  o Clarify whether neonatal mortality refers to deaths within 30 days or 28 days. The sentence suggests both: “Both of these sources use the standard DHS calculation for NMR based on neonatal deaths in the first month of life, or 30 days.” — verify accuracy. | - Rephrased the sentences as “NMR is a key indicator published in the final reports of the Nepal Demographic and Health Survey (NDHS) and made available through the STATcompiler platform”  -The study used 28 days as of WHO definition although the NDHS report used the defining of neonatal mortality up to 30 days. | Page #8, lines #8-#9  Page #8, lines #8-13 |
| 6. 7. 8. Page 7 – Ethical Considerations: o Clarify whether a separate ethical approval was obtained. If not, suggest writing that the NDHSs obtained ethical approvals from the NHR and a separate approval was not sought for this study based on secondary analysis of the NDHSs. | NDHSs obtained ethical approvals from the Nepal Health Research Council and a separate approval was not sought for this study as it is based on secondary analysis of the NDHSs | Page #10, lines #19-20 |
| 9. Page 8: o Expand ND (Neonatal Deaths) at first use. | Revised as suggested | Page #11, Line #6 |
| 10. Page 14: • The sentence is unclear: “While the association between ANC care and background variables is explored in Chapter 9 of the final report for both the 2016 and 2022 surveys, detailed articulation of these pathways is constrained by low statistical power in this analysis.” o Which report and which chapter are being referenced? Please clarify | Revised and cited the reference 5, and 6. We have referred the NDHS final reports of 2016 and 2022 | Page #16, 19 |
| 11. Page 16: • Consider simplifying the description of federalism. For example, clarify where municipalities belong within the three tiers (federal, provincial, local). | Clarified as federal, provincial, and local levels. | Page #18, lines #1, 4, 5 |
| 12. Page 17: • Recommend comparing NMR with the SDG 2030 target, rather than the NeNAP target, for consistency and relevance to international readers. | Revised as SDG 2030 target | Page #19, line #2 |
| 13. Page 19: • The phrase “maternal and husband” should likely be revised to “maternal and paternal.” | Revised | Page #20, line #19 |
| 14. Page 20: • Expand HeMG (Health Mothers’ Group) at first use. | Revised | Page #21, lines #14 |
| 15. Conclusions and Key Recommendations: • This section reads like Conclusions only. Recommendations can go into the Discussion section. Suggest strengthening this section by linking conclusions more explicitly to SDG targets. | Revised as suggested. “Achieving the SDG target of an NMR of 12 per 1,000 live births will require focused efforts on addressing equity gaps, enhancing maternal education, and improving access to skilled birth attendants at the time of delivery. Further, comprehensive newborn-focused research is needed to identify the underlying causes of neonatal mortality across diverse population”. | Page #23, lines #13-16 |
| 16. Study Limitations: • Recommend including a brief discussion of the limitations of the study. | Included the limitation about analysis that is not possible with power and also service utilization factors from mothers’ perspective | Page #22, lines #20-23  Page #23, Lines #3-4 |
| 17. Results and Discussion: • Consider shortening these sections to maintain the reader’s engagement. | We shortened the results and discussion section | Results and discussion |
| 18. Table 2: • Suggest replacing the term “respondent” with more appropriate terminology. • Clarify if “husband” refers to “father” in the context of the study. | Revised as suggested. | Table # 2 |
| 19. Figures: • Figures 1 and 2 are blurred and difficult to read — suggest improving image resolution. • Figure 3: Confirm if the “Late Neonatal Period” is 0–28 days or 0–27 days. Please verify. • Ensure consistent font size and type across all figures and graphs. | Late neonatal period is 7-27 days complete is considered as 28 days.  Revised all figures with fonts and clarity. | Figure 3 and all |
